# Mepce Restrains Ferroptosis in Prostate Cancer Through 7SK–P‐TEFb Pause Control

**DOI:** 10.1002/advs.76958

**Published:** 2026-08-05

**Authors:** Tongtong Zhang, Xiangyang Zhan, Jiexiang Zhang, Mingyue Tan, Chuanmin Chu, Guanqun Ju, Xinyu Zhai, Jianyi Gu, Huirong Zhu, Dongliang Xu

**Affiliations:** ^1^ Urology Centre Shuguang Hospital Affiliated to Shanghai University of Traditional Chinese Medicine Shanghai China; ^2^ Surgical Institute of Integrative Medicine Shuguang Hospital Affiliated to Shanghai University of Traditional Chinese Medicine Shanghai China; ^3^ Surgical Institute Shuguang Hospital Affiliated to Shanghai University of Traditional Chinese Medicine Shanghai China; ^4^ Shanghai Key Laboratory of Traditional Chinese Clinical Medicine Shanghai China; ^5^ Department of Medical Oncology Shuguang Hospital Shanghai University of Traditional Chinese Medicine Shanghai China

**Keywords:** 7SK snRNP, ferroptosis, GPX4 inhibition, MEPCE, prostate cancer

## Abstract

Ferroptosis can be therapeutically induced through GPX4 inhibition, but the mechanisms that determine ferroptosis sensitivity in prostate cancer remain incompletely understood. Here, we combined genome‐wide CRISPR screening under graded GPX4 inhibition with mechanistic perturbation–rescue experiments and validation in xenograft and immunocompetent syngeneic prostate cancer models. We identified MEPCE loss as a conserved sensitizer to ferroptosis. Mechanistically, MEPCE depletion destabilized RN7SK and disrupted the HEXIM1–P‐TEFb complex, consistent with CDK9 release and enhanced RNA polymerase II Ser2 phosphorylation, thereby promoting transcriptional elongation. This state accelerated activation of a NRF2/ARE stress program encompassing antioxidant defense and iron mobilization. Within this program, HMOX1 emerged as a kinetically sensitive effector whose rapid induction promoted labile iron accumulation and lipid peroxidation under GPX4 inhibition. In vivo, MEPCE suppression impaired tumor growth, enhanced the antitumor efficacy of ferroptosis induction, and was associated with pharmacodynamic markers of ferroptosis that were partially reversed by ferroptosis‐ or iron‐targeted rescue. Together, these findings identify a transcriptional pause‐control mechanism that links elongation dynamics to ferroptosis susceptibility and nominate the MEPCE–RN7SK–P‐TEFb axis as a potential therapeutic target for sensitizing prostate cancer to ferroptosis.

## Graphical abstract

Genome‐wide CRISPR screening identifies MEPCE as a pause‐control regulator that restrains ferroptosis in prostate cancer. MEPCE loss releases P‐TEFb/CDK9 from 7SK snRNP control, enhances RNAPII Ser2 phosphorylation, and accelerates NRF2‐HMOX1‐driven labile iron accumulation and lipid peroxidation, revealing a transcriptional kinetic gate that lowers the ferroptosis threshold.

## 1 Introduction

Ferroptosis is an iron‐dependent, lipid peroxidation–driven form of cell death that can be engaged therapeutically in cancer, including prostate cancer, by perturbing antioxidant defenses and membrane redox control [1]. Pharmacologic inhibition of GPX4 provides a direct route to trigger ferroptotic stress, yet tumor cells frequently buffer this insult, producing heterogeneous sensitivity that limits translational leverage [2]. A practical barrier is that many determinants of ferroptosis sensitivity are context‐dependent and can differ across cellular backgrounds and in vivo microenvironments, complicating the identification of robust, actionable control points [3]. Defining tumor‐intrinsic regulators that set the ferroptosis threshold, and establishing how these regulators connect to measurable pharmacodynamic endpoints, remains a central requirement for rational strategy design [4, 5].

This challenge is particularly relevant in prostate cancer, where therapeutic resistance and state plasticity increasingly expose dependencies on stress adaptation rather than on a single dominant signaling node [6]. Although androgen receptor pathway blockade has transformed the treatment landscape, advanced prostate tumors frequently evolve adaptive programs that preserve viability under metabolic, oxidative and therapeutic stress [7, 8]. These adaptations are likely to be especially important in aggressive and therapy‐refractory states, where redox buffering, iron handling and membrane lipid metabolism can shape whether ferroptotic damage is contained or propagated [9]. At the same time, prostate cancer is marked by substantial inter‐model and inter‐tumor heterogeneity, raising the possibility that ferroptosis sensitivity is not determined by one downstream effector alone, but instead by upstream regulatory states that tune the kinetics and amplitude of stress responses [10]. Identifying such upstream gates is therefore of both mechanistic and translational interest in this disease context.

Mechanistically, ferroptosis execution reflects the balance between lipid peroxide generation, iron availability, and the capacity of stress‐responsive transcriptional programs to remodel redox and iron‐handling states [11]. Stress signaling pathways, including NRF2‐dependent transcription, can reconfigure antioxidant and heme/iron circuits and thereby influence ferroptotic outcomes, but the kinetic and regulatory constraints that govern these responses are incompletely resolved in tumor cells [12]. This question has particular resonance in prostate cancer, where oxidative stress adaptation, lineage plasticity and therapy‐associated remodeling of cellular state may all influence how rapidly cytoprotective or iron‐mobilizing programs are deployed. Transcriptional control is not limited to factor occupancy at promoters and enhancers; promoter‐proximal pausing and elongation licensing shape the speed and magnitude with which stress genes are produced [13]. However, how pause control and elongation licensing interface with ferroptosis‐relevant outputs—such as labile iron accumulation and lipid peroxidation—has remained difficult to place into a causal chain in cancer models [14, 15].

A specific gap is the lack of an integrated framework that links an upstream, durable transcriptional gate to early ferroptosis chemical endpoints and to in vivo therapeutic interaction [16]. Existing approaches often emphasize endpoint viability or late transcriptional profiles, which can obscure the timing of stress‐gene induction and blur causality between transcriptional changes and ferroptosis chemistry [17]. This limitation is especially restrictive in prostate cancer, where tumor progression often unfolds through gradual adaptation to persistent therapeutic pressure and where tumor cells coexist with stromal, immune and metabolic influences that can modify redox state in vivo. In addition, determinants nominated in vitro may not persist in immunocompetent settings, where tumor growth, pharmacodynamic signals, and immune remodeling coexist [18]. Addressing this gap requires a strategy that simultaneously nominates conserved regulators across cell lines, tests mechanistic necessity through perturbation and rescue, and anchors the pathway to early, modality‐consistent readouts in tumors [19].

Here we combined genome‐wide CRISPR screening in prostate cancer cell lines with mechanistic interrogation of transcriptional pause control and multi‐model in vivo validation to define an upstream gate that couples elongation licensing to ferroptosis sensitivity. We used low‐ and high‐pressure GPX4 inhibition screens with ferroptosis‐specific rescue branches to nominate conserved regulators, prioritized MEPCE, and tested its role using targeted genetic perturbation, re‐expression rescue, and pharmacologic back‐perturbations of CDK9, NRF2, and HMOX1 across kinetic reporters, nascent transcription, and chemical endpoints of lipid peroxidation and labile iron. We then evaluated tumor fitness and therapeutic interaction in PC3‐luc xenografts and in an immunocompetent RM‐1 syngeneic model with an early pharmacodynamic window, complemented by ex vivo tumor slice assays and ferroptosis/iron rescue. The objective of this study was to establish whether a MEPCE‐linked pause‐control axis sets the ferroptosis threshold in prostate cancer by regulating CDK9‐dependent elongation and NRF2/HMOX1 kinetics, and to connect this mechanism to actionable pharmacodynamic markers across in vitro and in vivo models.

## 2 Results

### 2.1 MEPCE Suppression Primes Prostate Cancer Cells for Ferroptosis‐ and Iron‐Dependent Collapse

We established orthogonal, ferroptosis‐focused genome‐wide CRISPR screens to identify conserved sensitizers and resisters of GPX4 inhibition across prostate cancer contexts. PC3‐Cas9 and DU145‐Cas9 cells were transduced with a pooled genome‐wide sgRNA library, selected, expanded under conditions preserving library representation, and sampled at T0 for baseline genomic DNA profiling (Figure 1a). Screening arms included DMSO control, sublethal RSL3 to identify depletion‐based sensitizers, lethal RSL3 to capture enrichment‐based resisters, and a ferroptosis‐specificity branch in which RSL3_low was counterselected by Ferrostatin‐1 rescue (Figure 1a). This design enabled separation of sensitization from relative resistance while incorporating an internal modality control for ferroptotic cell death.

**FIGURE 1:**
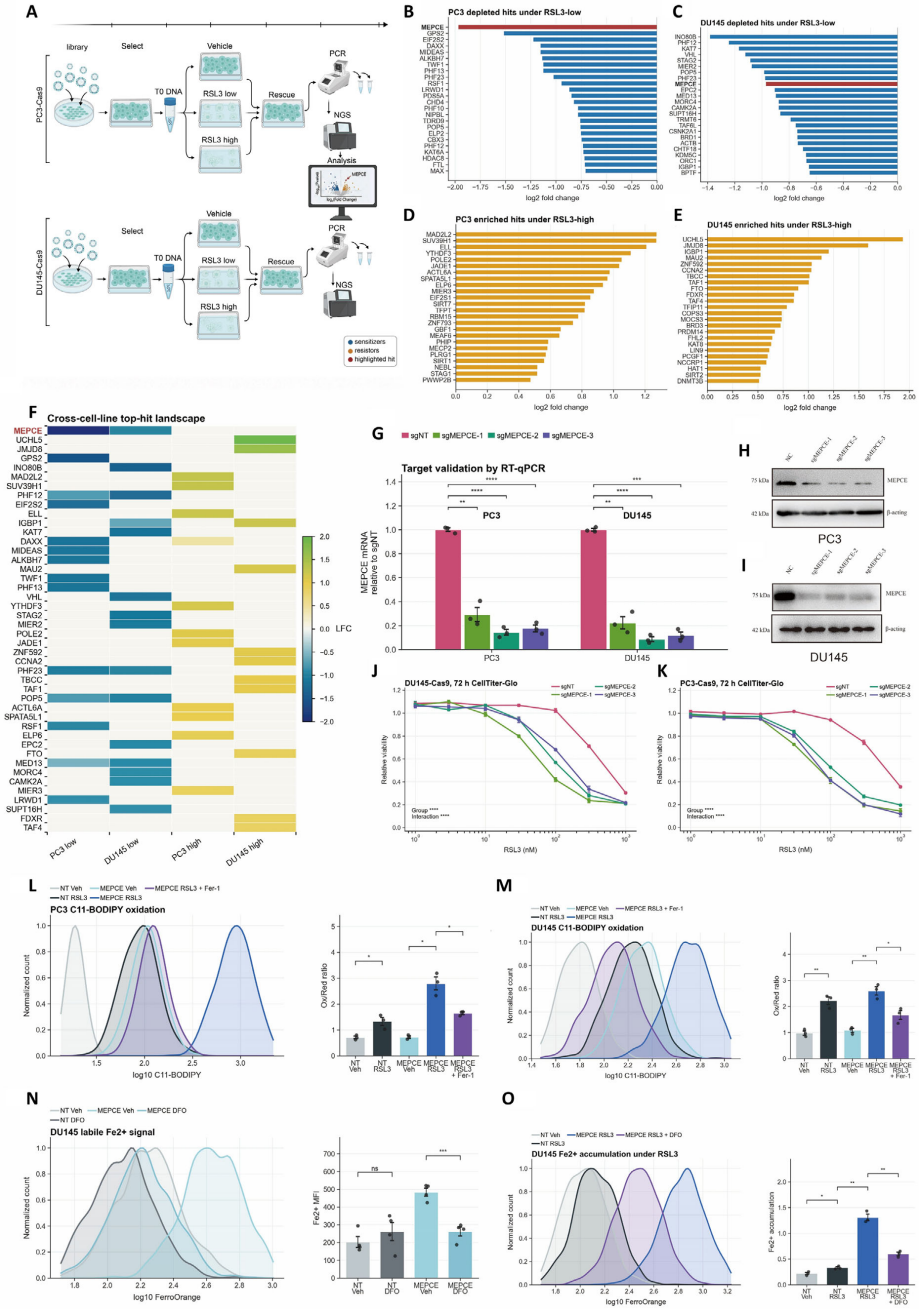
Genome‐wide CRISPR ferroptosis screens identify MEPCE loss as a conserved sensitizer of GPX4 inhibition in prostate cancer cells. (a) Schematic of pooled genome‐wide CRISPR knockout screening in PC3‐Cas9 and DU145‐Cas9 cells. Cells were screened under DMSO control, sublethal RSL3 (RSL3_low) to identify depletion‐based sensitizers, lethal RSL3 (RSL3_high) to identify enrichment‐based resistance factors, and RSL3_low combined with Ferrostatin‐1 to assess ferroptosis specificity, followed by sgRNA sequencing and gene‐level analysis. (b, c) Ranked depletion plots showing the top depleted genes under RSL3_low in PC3‐Cas9 (b) and DU145‐Cas9 (c); depletion indicates genes whose loss sensitized cells to GPX4 inhibition. (d, e) Ranked enrichment plots showing the top enriched genes under RSL3_high in PC3‐Cas9 (d) and DU145‐Cas9 (e); enrichment indicates genes whose loss conferred relative resistance under high‐pressure GPX4 inhibition. (f) Integrated heat map summarizing gene‐level effects across PC3_low, DU145_low, PC3_high, and DU145_high screening conditions. Color indicates relative depletion or enrichment log‐fold change across conditions. (g) RT–qPCR validation of MEPCE knockdown in PC3‐Cas9 and DU145‐Cas9 cells transduced with independent sgMEPCE constructs; summary metric, relative MEPCE mRNA normalized to sgNT. (h, i) Immunoblot validation of MEPCE protein reduction in PC3‐Cas9 and DU145‐Cas9 cells expressing sgMEPCE; loading control as indicated. (j, k) 72 h CellTiter‐Glo dose–response curves to RSL3 in PC3‐Cas9 and DU145‐Cas9 cells comparing sgNT and sgMEPCE backgrounds; summary metric, relative viability normalized to vehicle‐treated controls. (l, m) C11‐BODIPY flow cytometry in PC3‐Cas9 and DU145‐Cas9 cells treated with DMSO, RSL3, or RSL3 plus Ferrostatin‐1 in sgNT or sgMEPCE backgrounds; summary metric, lipid‐peroxidation oxidation ratio derived from oxidized versus reduced C11‐BODIPY signal. (n, o) FerroOrange measurement of labile Fe^2+^ in DU145‐Cas9 cells in sgNT and sgMEPCE backgrounds at baseline or after short‐term RSL3 treatment with or without DFO; summary metrics, fluorescence intensity and high‐Fe^2+^ fraction. Significance is indicated by asterisks: ns, not significant; ^*^ *p* < 0.05; ^**^ *p* < 0.01; ^***^ *p* < 0.001; ^****^ *p* < 0.0001.

Gene‐level ranking nominated MEPCE as a candidate cross‐context sensitizer and defined complementary sensitizer and resister landscapes. In PC3 and DU145 cells exposed to RSL3_low, depletion‐based analyses using MAGeCK and drugZ identified top depleted genes consistent with ferroptosis sensitizers, with MEPCE emerging in the same direction across both models (Figure 1b,c). Under lethal selection pressure, RSL3_high screens identified enriched genes consistent with relative resistance in both cell lines (Figure 1d,e). Integrative analysis across PC3_low, DU145_low, PC3_high and DU145_high further highlighted a subset of robust dependencies, providing a rationale to prioritize MEPCE for downstream validation (Figure 1f).

Independent sgRNAs efficiently suppressed MEPCE expression and revealed a reproducible RSL3‐sensitization phenotype in both prostate cancer models. In PC3‐Cas9 and DU145‐Cas9 cells, multiple sgMEPCE reagents reduced MEPCE mRNA abundance relative to sgNT controls, and immunoblotting confirmed effective depletion of MEPCE protein in both lines (Figure 1g–i). Functionally, 72 h CellTiter‐Glo dose–response assays demonstrated enhanced sensitivity to RSL3 upon MEPCE disruption in both models, manifested as a consistent leftward shift in the dose–response curves across independent sgRNAs (Figure 1j,k). These data indicated that MEPCE constrains cellular vulnerability to GPX4 inhibition and warranted ferroptosis‐directed rescue and biochemical validation.

Chemical rescue and orthogonal biochemical readouts further constrained the phenotype caused by MEPCE loss to iron‐dependent lipid peroxidation, consistent with ferroptosis. Consistent with a ferroptotic endpoint, C11‐BODIPY assays showed that RSL3‐induced lipid peroxidation was further augmented in sgMEPCE cells, whereas Ferrostatin‐1 suppressed this oxidation signal in both PC3 and DU145 cells (Figure 1l,m). In DU145 cells, FerroOrange flow cytometry revealed increased Fe^2^^+^ signal in the sgMEPCE background, which was attenuated by DFO; under short‐term RSL3 challenge, Fe^2^^+^ accumulation further increased and remained more pronounced in sgMEPCE cells, again with suppression by DFO (Figure 1n,o).

To assess the clinical relevance of MEPCE in prostate cancer, we first examined its expression across clinicopathological strata in public TCGA prostate cancer datasets. MEPCE expression was modestly but consistently increased in tumors with more advanced pathological and clinical features, including higher pathologic T stage, nodal involvement and metastatic status, supporting an association between MEPCE expression and disease progression (Figure S1a–c). We then examined whether MEPCE abundance in clinical tumor specimens was related to the elongation‐associated RNAPII Ser2 phosphorylation axis implicated by our mechanistic studies. Immunoblot analysis of representative human prostate tumor samples showed that cases with lower MEPCE protein abundance displayed higher pSer2‐Pol II signal (Figure S1d). Thus, although MEPCE expression is clinically retained or elevated in prostate cancer, its relative reduction within tumors is associated with increased RNAPII Ser2 phosphorylation, consistent with our model that MEPCE restrains CDK9‐dependent transcriptional elongation in prostate cancer cells. To further assess the robustness of the screening results beyond MEPCE, we selected additional top‐ranked candidates from both the depletion‐ and enrichment‐based arms for targeted validation in PC3‐Cas9 and DU145‐Cas9 cells. BRPF3 was prioritized as a representative shared enrichment hit, whereas KAT6A, PHF12 and PHF23 were selected as representative shared depletion hits on the basis of cross‐cell‐line reproducibility and rank position in the original screens. Independent sgRNAs efficiently reduced BRPF3, KAT6A, PHF12 and PHF23 expression in both DU145‐Cas9 and PC3‐Cas9 cells, as confirmed by RT–qPCR (Figure S2a–h). Functionally, BRPF3 disruption conferred partial resistance to GPX4 inhibition, as reflected by a rightward shift of the RSL3 dose–response curves and increased viability relative to sgNT controls in both cell lines (Figure S2i, j). By contrast, disruption of KAT6A, PHF12 or PHF23 increased sensitivity to RSL3, producing consistent leftward shifts in the dose–response curves in both DU145‐Cas9 and PC3‐Cas9 cells (Figure S2k–p). Among these validated depletion hits, PHF12 and PHF23 showed the strongest sensitization phenotype, whereas KAT6A displayed a reproducible but comparatively more moderate effect.

### 2.2 MEPCE Loss Releases CDK9 to Accelerate NRF2/HMOX1 Stress Transcription Through 7SK Pause Control

We next integrated mechanistic perturbation analyses to link MEPCE to 7SK‐dependent pause control and downstream ferroptosis‐associated stress outputs. A working model posited that MEPCE sustains the inhibitory 7SK snRNP state to restrain P‐TEFb release, whereas MEPCE loss promotes CDK9‐dependent phosphorylation of RNAPII on Ser2, amplifies NRF2/ARE signaling, accelerates HMOX1 induction and ultimately enhances iron‐dependent lipid peroxidation, thereby lowering the ferroptotic threshold (Figure 2a). We therefore tested this cascade stepwise, from 7SK abundance and stability, to inhibitory‐complex integrity and elongation‐associated phosphorylation, and finally to stress‐gene induction kinetics and NRF2‐dependent transcriptional output (Figure 2b–m).

**FIGURE 2:**
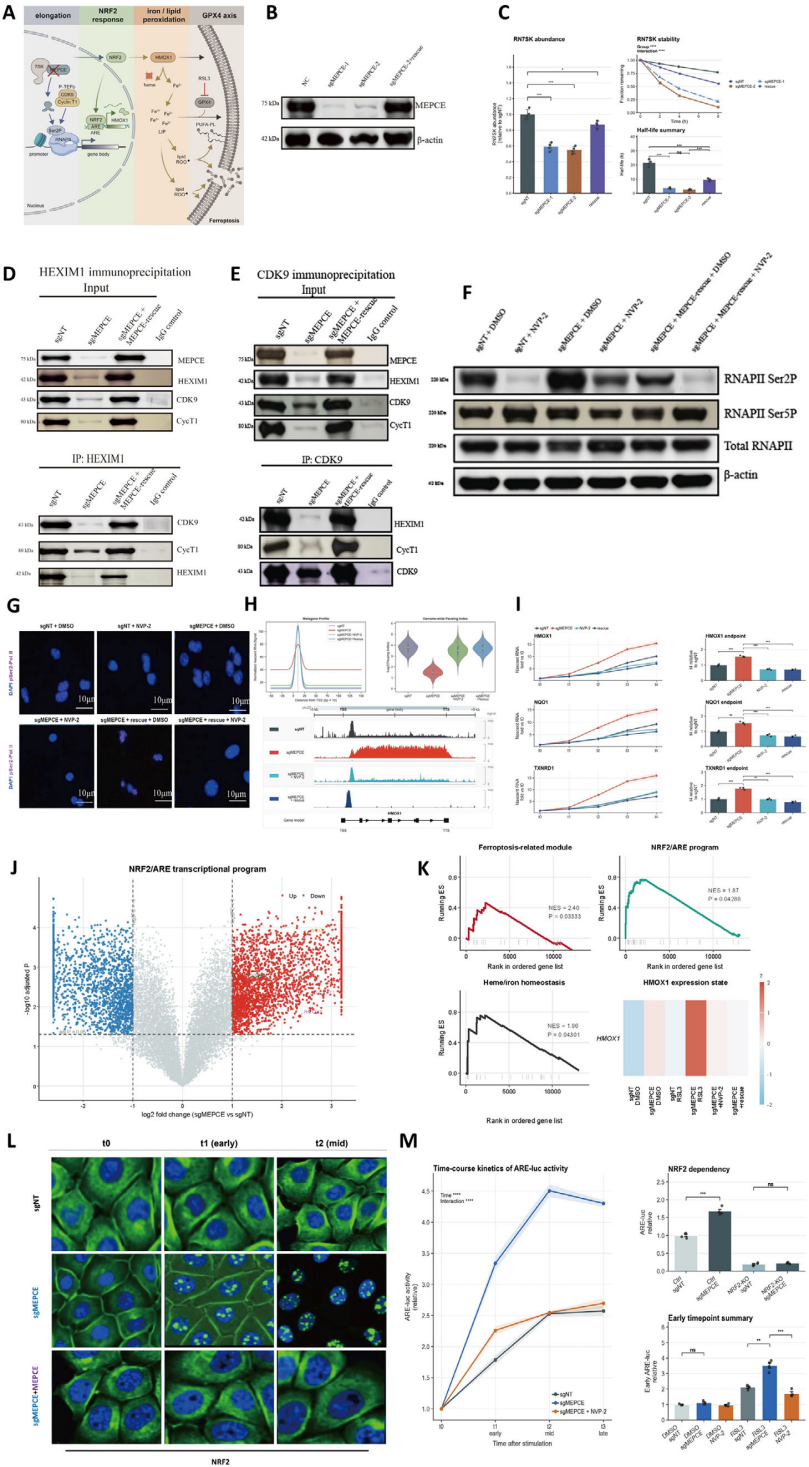
MEPCE loss destabilizes 7SK snRNP and increases CDK9‐dependent elongation and NRF2‐responsive transcription. (a) Working model linking MEPCE‐dependent 7SK snRNP restraint of P‐TEFb/CDK9 to RNAPII Ser2 phosphorylation, NRF2/ARE activation, iron handling, lipid peroxidation, and ferroptosis sensitivity. (b) Immunoblot of MEPCE in sgNT, sgMEPCE, and MEPCE‐rescue cells confirming target suppression and rescue. (c) RT–qPCR measurement of RN7SK steady‐state abundance and RN7SK decay after transcriptional shutoff in sgNT, sgMEPCE, and MEPCE‐rescue cells; U6 served as a control RNA. Summary metric, relative RNA abundance normalized to control. (d, e) Co‐immunoprecipitation analysis of HEXIM1–CDK9/Cyclin T1 association (d) and reciprocal CDK9–HEXIM1 association (e) in sgNT, sgMEPCE, and rescue cells; summary metric, co‐immunoprecipitated protein normalized to immunoprecipitated bait. (f) Immunoblot of RNAPII Ser2P, RNAPII Ser5P, and total RNAPII in sgNT, sgMEPCE, and MEPCE‐rescue cells treated with or without NVP‐2; summary metric, phospho‐RNAPII/total RNAPII ratio. (g) Single‐cell immunofluorescence measurement of nuclear pSer2‐RNAPII in sgNT, sgMEPCE, sgMEPCE plus NVP‐2, and rescue cells; summary metric, per‐cell nuclear fluorescence intensity distribution. (h) Nascent‐transcription profiling under sgNT, sgMEPCE, sgMEPCE plus NVP‐2, and MEPCE‐rescue conditions. (i) Nascent RNA time‐course measurements of HMOX1, NQO1 and TXNRD1 in sgNT, sgMEPCE, sgMEPCE plus NVP‐2, and rescue cells; summary metric, relative nascent transcript abundance over time. (j) Volcano plot of differential gene expression in sgMEPCE vs. sgNT cells. (k) Gene set enrichment analyses for NRF2/ARE, heme/iron, and ferroptosis‐related pathways under the indicated conditions; summary metric, normalized enrichment score. (l) Time‐resolved immunofluorescence measurement of NRF2 nuclear accumulation in sgNT, sgMEPCE, and rescue cells; summary metric, nuclear/cytoplasmic NRF2 ratio. (m) ARE‐luciferase kinetics in sgNT, sgMEPCE, sgMEPCE plus NVP‐2, and NRF2‐knockout cells; summary metric, firefly/Renilla luciferase ratio over time. Significance is indicated by asterisks: ns, not significant; ^*^ *p* < 0.05; ^**^ *p* < 0.01; ^***^ *p* < 0.001; ^****^ *p* < 0.0001.

Immunoblotting confirmed efficient depletion of MEPCE by sgMEPCE and restoration of MEPCE expression in rescue cells (Figure 2b). MEPCE depletion destabilized RN7SK and weakened the HEXIM1–P‐TEFb inhibitory complex, consistent with release of CDK9 activity. Steady‐state RN7SK abundance was reduced in sgMEPCE cells relative to sgNT controls, and transcriptional shut‐off chase experiments revealed accelerated RN7SK decay and a shortened half‐life, whereas the control small RNA U6 remained largely unchanged (Figure 2c). In HEXIM1 CoIP, MEPCE loss reduced co‐precipitation of CDK9 and Cyclin T1, whereas MEPCE re‐expression restored these interactions; reciprocal CoIP of CDK9 yielded a concordant reduction in CDK9–HEXIM1 association in sgMEPCE cells, again reversible by MEPCE rescue (Figure 2d,e). These perturbations predicted increased CDK9‐dependent elongation licensing, which we next examined at the level of RNAPII phosphorylation.

Consistent with this model, MEPCE loss increased global RNAPII Ser2 phosphorylation in a CDK9‐dependent manner. Immunoblotting showed that sgMEPCE increased RNAPII Ser2 phosphorylation relative to total RNAPII, whereas NVP‐2 strongly suppressed this increase and MEPCE re‐expression shifted pSer2 levels back toward control; by contrast, changes in pSer5 were comparatively limited (Figure 2f). Single‐cell immunofluorescence for nuclear pSer2 recapitulated these findings, revealing a population‐wide shift toward higher nuclear pSer2 signal in sgMEPCE cells, with suppression by NVP‐2 and reversal by MEPCE rescue (Figure 2g). These data support a perturbation‐and‐rescue chain linking MEPCE loss to CDK9‐dependent Ser2 phosphorylation and prompted direct assessment of promoter‐proximal pausing and elongation outputs at the nascent transcriptional level.

Nascent‐transcription profiling further indicated reduced promoter‐proximal pausing and enhanced productive elongation following MEPCE loss. Metagene analysis showed increased gene‐body nascent transcription signal in sgMEPCE cells, and genome‐wide pausing‐index distributions shifted toward lower values, consistent with increased pause release. Both NVP‐2 treatment and MEPCE re‐expression partially normalized these changes (Figure 2h). At a representative HMOX1 locus, sgMEPCE cells showed stronger gene‐body nascent transcription and an elongation‐favored signal pattern, which was attenuated by CDK9 inhibition or by MEPCE rescue (Figure 2h). Gene‐specific nascent RNA time‐course assays further confirmed accelerated induction of NRF2/stress‐responsive transcripts, including HMOX1, NQO1 and TXNRD1 (Figure 2i).

Transcriptomic and reporter‐based analyses indicated that MEPCE loss accelerates a mixed NRF2/ARE stress program rather than a uniformly pro‐ferroptotic transcriptional state. Differential expression analysis comparing sgMEPCE with sgNT cells identified increased expression of several NRF2/ARE and oxidative‐stress‐associated genes, including HMOX1, NQO1, SLC7A11 and TXNRD1 (Figure 2j). Because these targets include both antioxidant‐buffering genes and heme/iron‐handling genes, we interpreted this response as a composite stress‐adaptive program whose ferroptosis outcome depends on the balance and timing of individual downstream modules. Gene‐set enrichment analysis further showed increased enrichment of NRF2/ARE, heme metabolism, iron‐homeostasis and ferroptosis‐related pathways in sgMEPCE cells; these enrichments were reduced by NVP‐2 treatment and by MEPCE rescue, and were more evident under RSL3 stress than under DMSO control (Figure 2k). In line with these program‐level changes, time‐resolved immunofluorescence revealed earlier and/or stronger nuclear accumulation of NRF2 in sgMEPCE cells, whereas MEPCE re‐expression shifted NRF2 translocation kinetics back toward the control state (Figure 2l). ARE‐luciferase assays likewise showed a faster kinetic rise and a higher activity trend in sgMEPCE cells, suppression of this amplification by NVP‐2, and marked reduction of reporter activity upon NRF2 knockout, thereby establishing NRF2 dependence of the readout and supporting CDK9‐mediated elongation as a determinant of both the magnitude and timing of NRF2 transcriptional output (Figure 2m).

### 2.3 MEPCE Loss Couples Accelerated HMOX1 Output To Iron‐Dependent Ferroptotic Endpoints

MEPCE depletion accelerated the onset and increased the rate of HMOX1 transcriptional output, an effect reversed by MEPCE re‐expression. Time‐resolved reporter assays showed that sgMEPCE shifted HMOX1‐luc induction to an earlier time point and increased the rate of signal accumulation relative to sgNT controls, whereas MEPCE rescue restored the induction trajectory toward baseline (Figure 3a). Comparison at early time points further showed that sgMEPCE increased the initial amplitude of HMOX1‐luc activity, with rescue attenuating this early amplification (Figure 3b). Consistent with a primary effect on transcriptional production rather than delayed downstream accumulation, nascent HMOX1 RNA rose more rapidly in the sgMEPCE background, and this early induction was blunted by CDK9 inhibition or NRF2 loss (Figure 3c). A kinetic summary metric recapitulated the same ordering across conditions, showing that sgMEPCE shortened the time to half‐maximal induction, whereas CDK9 inhibition, NRF2 deficiency and MEPCE rescue restored slower kinetics (Figure 3d). Together, these data identify HMOX1 as a stress‐responsive kinetic gate downstream of MEPCE and place its rapid induction under joint control of CDK9 and NRF2. Initial locus‐focused assays indicated that altered HMOX1 output was accompanied by only modest changes in NRF2 occupancy, whereas RNAPII Ser2 phosphorylation across the HMOX1 gene body increased more clearly in sgMEPCE cells and was reduced by CDK9 inhibition or MEPCE rescue (Figure 3e–g).

**FIGURE 3:**
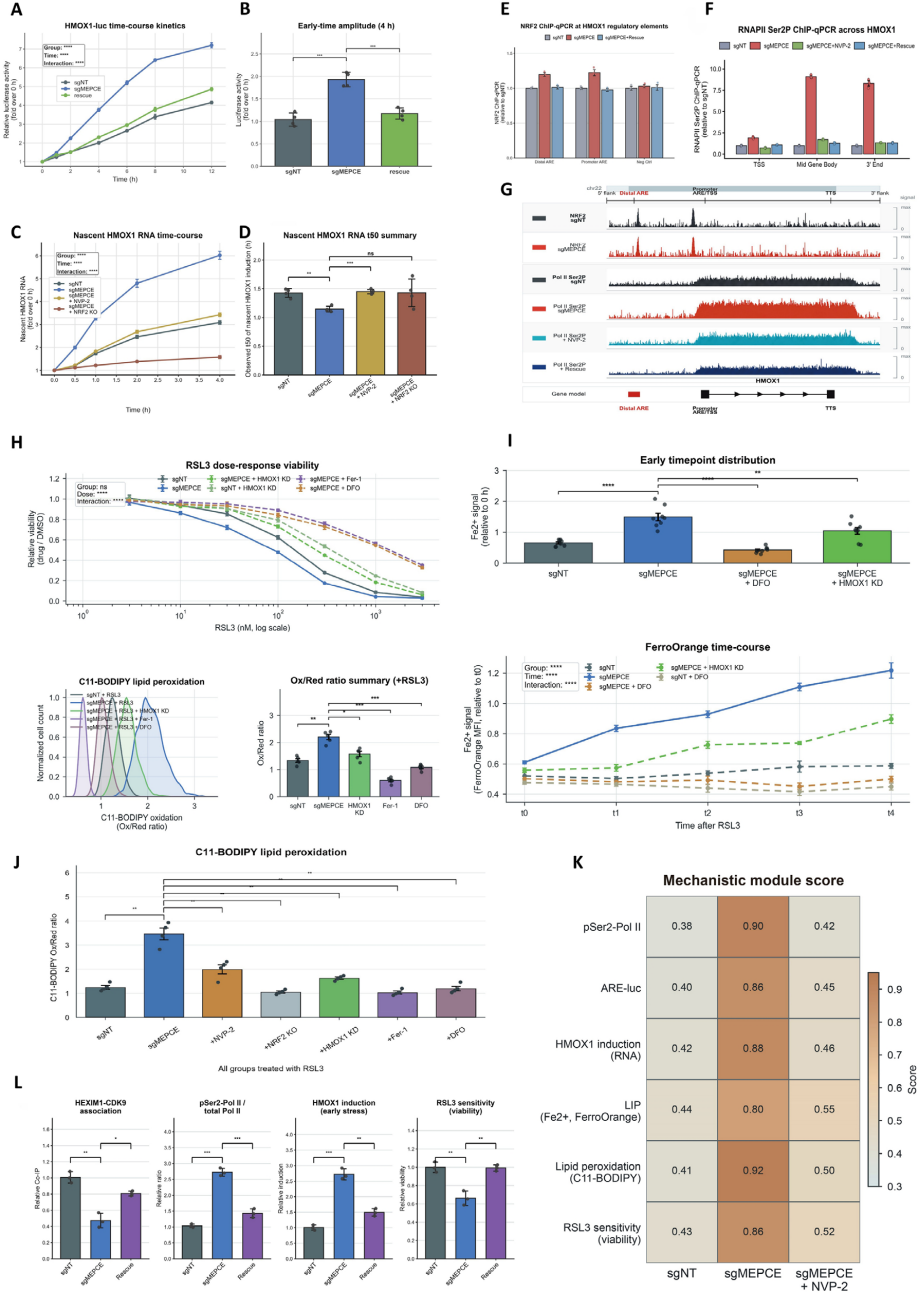
HMOX1 is a prominent kinetic effector linking elongation control to labile iron, lipid peroxidation, and RSL3 sensitivity. (a, b) HMOX1‐luciferase time‐course measurements in sgNT, sgMEPCE, and MEPCE‐rescue cells; summary metric in b, early reporter amplitude at the indicated time point. (c, d) Nascent HMOX1 RNA time course in sgNT and sgMEPCE cells with or without NVP‐2, NRF2 knockout, or rescue; summary metric in d, t50, defined as time to half‐maximal induction. (e) NRF2 occupancy at the HMOX1 promoter and distal enhancer AREs in sgNT, sgMEPCE, and rescue cells; summary metric, enrichment relative to control region/input. (f) Comparison of NRF2 occupancy and Pol II Ser2P enrichment across TSS‐proximal and gene‐body regions of HMOX1 in sgNT, sgMEPCE, sgMEPCE plus NVP‐2, and rescue cells; summary metric, normalized locus enrichment. (g) Representative locus tracks for NRF2 and Pol II Ser2P at HMOX1 in sgNT and sgMEPCE cells. (h) RSL3 dose–response viability curves in PC3‐Cas9 cells under sgNT and sgMEPCE conditions with HMOX1 knockdown, HO‐1 inhibition, Ferrostatin‐1, or DFO; matched C11‐BODIPY oxidation measurements were performed in the same perturbation framework. Summary metrics, relative viability and oxidation ratio. (i) FerroOrange time‐course measurement of labile Fe^2+^ after stress in sgNT, sgMEPCE, sgMEPCE plus DFO, and sgMEPCE plus HMOX1 knockdown cells; summary metric, fluorescence intensity over time. (j) Summary of C11‐BODIPY oxidation across CDK9 inhibition, NRF2 knockout, HMOX1 suppression, Ferrostatin‐1, or DFO conditions; summary metric, oxidation ratio. (k) Integrated matrix summarizing pSer2‐RNAPII, ARE‐luciferase output, HMOX1 induction, labile iron, lipid peroxidation. (l) Consolidated comparison of HEXIM1–CDK9 association, pSer2‐RNAPII, early HMOX1 induction, and RSL3 sensitivity in sgNT, sgMEPCE, and MEPCE‐rescue cells. Significance is indicated by asterisks: ns, not significant; ^*^ *p* < 0.05; ^**^ *p* < 0.01; ^***^ *p* < 0.001; ^****^ *p* < 0.0001.

Functional perturbation experiments positioned CDK9, NRF2 and HMOX1 upstream of iron accumulation, lipid peroxidation and ferroptotic sensitivity in the sgMEPCE state. In PC3 cells, sgMEPCE increased sensitivity to RSL3, and suppression of HMOX1, either by knockdown or by pharmacological inhibition of HO‐1 activity, attenuated this sensitization; canonical ferroptosis inhibitors and iron‐targeting interventions similarly opposed the phenotype (Figure 3h). Within the same framework, sgMEPCE increased stress‐induced lipid peroxidation as measured by C11‐BODIPY oxidation, and this increase was reduced by HO‐1 inhibition, ferroptosis suppression or iron chelation (Figure 3h). Time‐resolved Fe2+ measurements further showed that MEPCE loss accelerated and augmented accumulation of the labile iron pool after stress, whereas this increase was diminished by iron chelation and by suppression of HMOX1, thereby linking the HMOX1 gate to iron availability (Figure 3i). A consolidated summary of lipid peroxidation across perturbations further placed CDK9 inhibition and NRF2 loss upstream of lipid oxidation, alongside HMOX1 suppression and ferroptosis‐ or iron‐directed interventions, reinforcing a hierarchical pathway from transcriptional control to biochemical execution (Figure 3j). A multi‐readout matrix summarized the directional coherence of this cascade, with sgMEPCE driving increased RNAPII Ser2 phosphorylation, enhanced ARE output, accelerated HMOX1 induction, elevated labile iron, increased lipid peroxidation and heightened RSL3 sensitivity, all of which were broadly attenuated by CDK9 inhibition (Figure 3k). Finally, coordinated reversibility by MEPCE rescue across inhibitory‐complex state, elongation‐associated phosphorylation, early HMOX1 induction and RSL3 sensitivity supported a linked mechanistic chain rather than a set of parallel, independent effects (Figure 3l).

### 2.4 Quantitative Kinetic and Locus‐Level Analyses Define CDK9‐Dependent Pause Release as the Major Driver of HMOX1 OUTPUT

MEPCE depletion advanced and sharpened the temporal induction of an HMOX1 reporter, and this kinetic shift was reversible upon MEPCE re‐expression. Across a time course, sgMEPCE shifted HMOX1‐luc activation to an earlier onset and increased the rate of signal rise relative to sgNT controls, whereas MEPCE rescue restored the trajectory toward the control pattern (Figure 4a). Quantitative kinetic features extracted from curve fitting recapitulated these changes, showing that sgMEPCE compressed onset and mid‐response timing while increasing the steepness of induction, with rescue reversing these parameters toward baseline (Figure 4b). Here, we use the term “kinetic gate” to refer operationally to a downstream stress‐response node whose induction timing and rate are altered by upstream perturbation and whose altered dynamics track with downstream biochemical and phenotypic outputs.

**FIGURE 4:**
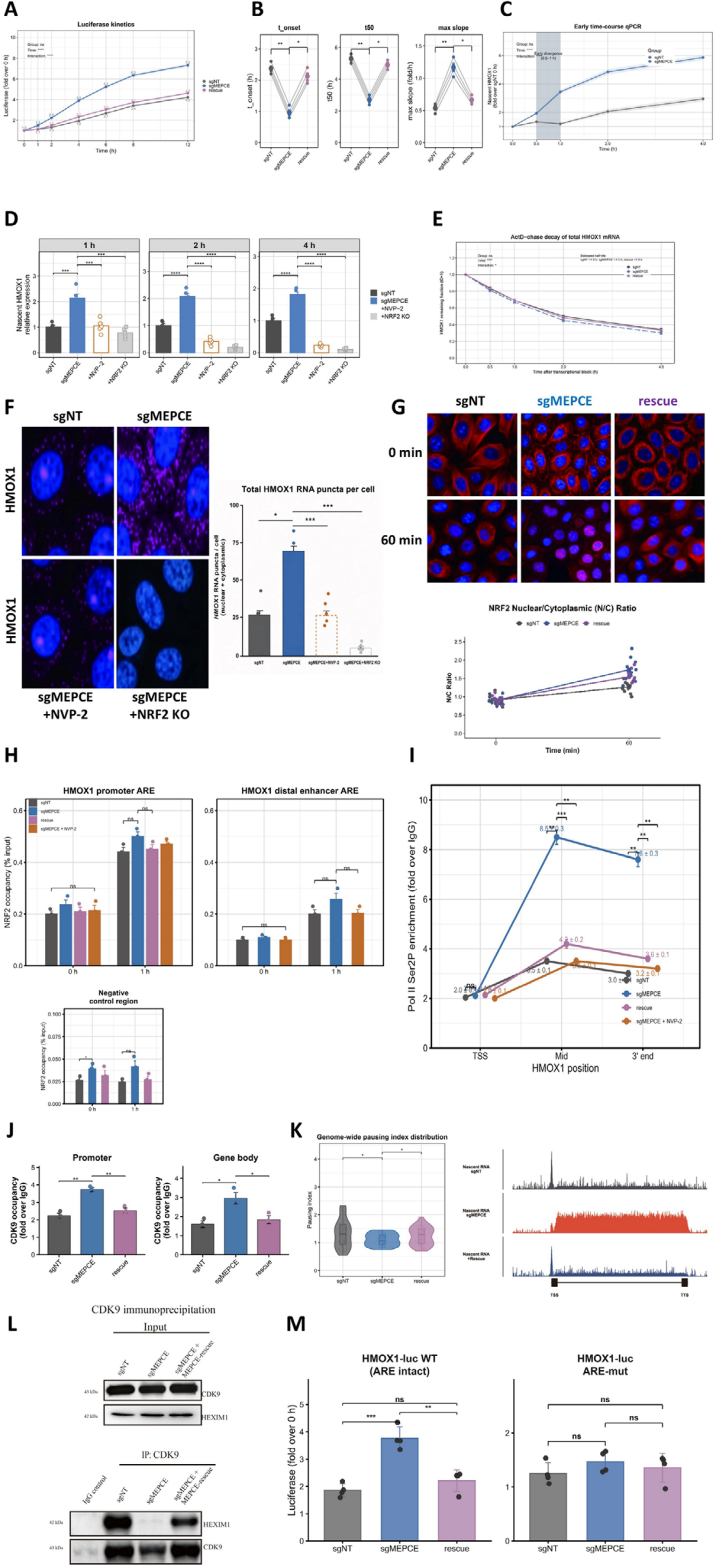
Quantitative kinetic and locus‐level analyses support CDK9‐dependent pause release as a major driver of accelerated HMOX1 output. (a, b) HMOX1‐luciferase time course and fitted kinetic parameters in sgNT, sgMEPCE, and MEPCE‐rescue cells; summary metrics, t_onset, t50, and maximal slope derived from fitted reporter curves. (c, d) Early nascent HMOX1 time course in sgNT, sgMEPCE, sgMEPCE plus NVP‐2, and sgMEPCE plus NRF2 knockout cells; summary metrics, integrated early output and discrete early time‐point abundance. (e) Actinomycin D chase of total HMOX1 mRNA in sgNT, sgMEPCE, and MEPCE‐rescue cells; summary metric, relative mRNA abundance normalized to time 0. (f) smFISH analysis of HMOX1 transcripts in sgNT, sgMEPCE, sgMEPCE plus NVP‐2, and sgMEPCE plus NRF2 knockout cells; summary metrics, puncta per cell and fraction of cells with active transcription sites. (g) Immunofluorescence measurement of NRF2 nuclear activation; summary metric, nuclear/cytoplasmic NRF2 ratio. (h) NRF2 occupancy at the HMOX1 promoter, distal enhancer AREs, and a negative‐control region under sgNT, sgMEPCE, rescue, and sgMEPCE plus NVP‐2 conditions; summary metric, normalized enrichment. (i) Pol II Ser2P enrichment across HMOX1 TSS, mid‐gene body, and 3′ end under sgNT, sgMEPCE, sgMEPCE plus NVP‐2, and rescue conditions; summary metric, normalized locus enrichment. (j) CDK9 occupancy at the HMOX1 promoter and gene body under sgNT, sgMEPCE, rescue, and NVP‐2 conditions; summary metric, normalized enrichment. (k) Genome‐wide pausing‐index distribution and HMOX1 locus tracks in sgNT, sgMEPCE, and rescue cells; pausing index was defined as promoter‐proximal signal divided by gene‐body signal. (l) CDK9 immunoprecipitation showing HEXIM1 co‐association in sgNT, sgMEPCE, and rescue cells; summary metric, co‐immunoprecipitated HEXIM1 normalized to immunoprecipitated CDK9. **m,** Wild‐type and ARE‐mutant HMOX1‐luciferase reporter activity in sgNT, sgMEPCE, and rescue cells; summary metric, normalized luciferase activity. Significance is indicated by asterisks: ns, not significant; ^*^ *p* < 0.05; ^**^ *p* < 0.01; ^***^ *p* < 0.001; ^****^ *p* < 0.0001.

Nascent transcription analyses placed this kinetic gate at the level of transcriptional production and linked it to CDK9 activity and NRF2. Early nascent HMOX1 time courses showed that sgMEPCE increased the initial accumulation of nascent HMOX1 RNA, whereas CDK9 inhibition and NRF2 loss reduced this early rise (Figure 4c,d). By contrast, actinomycin D chase experiments revealed closely overlapping decay kinetics of total HMOX1 mRNA across sgNT, sgMEPCE and rescue conditions, arguing against altered mRNA stability as the principal explanation for the increased output (Figure 4e). Thus, the accelerated HMOX1 response arises predominantly at the level of transcriptional production.

Single‐cell measurements supported broader and earlier HMOX1 transcriptional engagement together with faster NRF2 nuclear activation following MEPCE loss. smFISH analysis showed increased HMOX1 transcript puncta per cell and a higher fraction of cells harboring active transcription sites in sgMEPCE cells, with both effects attenuated by CDK9 inhibition or NRF2 loss (Figure 4f). Immunofluorescence likewise revealed increased nuclear‐to‐cytoplasmic NRF2 ratios after stimulation in sgMEPCE cells and a shift back toward baseline upon rescue (Figure 4g). These data placed accelerated NRF2 activation in temporal alignment with the HMOX1 kinetic shift.

Locus‐level chromatin and nascent transcription readouts implicated enhanced elongation licensing, rather than uniformly increased NRF2 occupancy, as the dominant mechanism underlying accelerated HMOX1 output. Measurements of NRF2 occupancy at ARE‐containing regulatory elements of HMOX1 showed only limited differences among sgNT, sgMEPCE and rescue conditions in most comparisons, while a negative control region remained unenriched (Figure 4h). By contrast, RNAPII Ser2 phosphorylation increased across the HMOX1 gene body in sgMEPCE cells and was reduced by CDK9 inhibition or by MEPCE rescue, consistent with enhanced elongation licensing driving more productive transcription through the locus (Figure 4i). CDK9 occupancy at HMOX1 was likewise increased in the sgMEPCE state and restored toward control by rescue (Figure 4j). At the genome‐wide level, pausing‐index distributions shifted toward lower values in sgMEPCE cells and reverted toward sgNT upon rescue, while HMOX1 locus tracks showed reduced promoter‐proximal pausing and increased gene‐body transcription under MEPCE loss (Figure 4k). Together, these data identify CDK9‐dependent pause release and elongation output as a principal mechanism governing the HMOX1 kinetic gate.

Upstream complex‐state and *cis*‐regulatory analyses further connected P‐TEFb release and NRF2–ARE dependence to this accelerated gate. Immunoprecipitation of CDK9 revealed reduced co‐association with HEXIM1 in sgMEPCE cells, whereas MEPCE rescue restored the CDK9–HEXIM1 interaction, consistent with release of P‐TEFb from its inhibitory complex state upon MEPCE depletion (Figure 4l). At the reporter level, an HMOX1 construct containing an intact ARE displayed stronger activity under sgMEPCE that was reduced by rescue, whereas mutation of the ARE markedly attenuated or abolished the sgMEPCE‐dependent difference (Figure 4m). These data therefore constrain the accelerated HMOX1 response to an NRF2–ARE‐dependent transcriptional mechanism operating in the context of enhanced P‐TEFb release and CDK9‐linked elongation control.

### 2.5 MEPCE Loss Restrains Tumor Fitness and Amplifies a Ferroptosis‐Linked Therapeutic Window In Vivo

We next established an in vivo framework to determine whether tumor‐intrinsic MEPCE suppression alters tumor fitness and engages the proposed transcriptional stress axis under baseline conditions. A schematic of the experimental design is shown in Figure 5a and outlines subcutaneous implantation of PC3‐luc cells expressing sgNT or sgMEPCE, volume‐balanced randomization, a vehicle‐only observation period, and endpoint collection for tumor burden and pharmacodynamic analyses. Under vehicle treatment, sgMEPCE tumors displayed slower growth than sgNT controls by serial caliper assessment (Figure 5b). At endpoint, excised tumors from the sgMEPCE group were visibly smaller and showed reduced tumor weight and overall burden, indicating that MEPCE suppression impairs tumor‐intrinsic fitness in vivo (Figure 5c).

**FIGURE 5:**
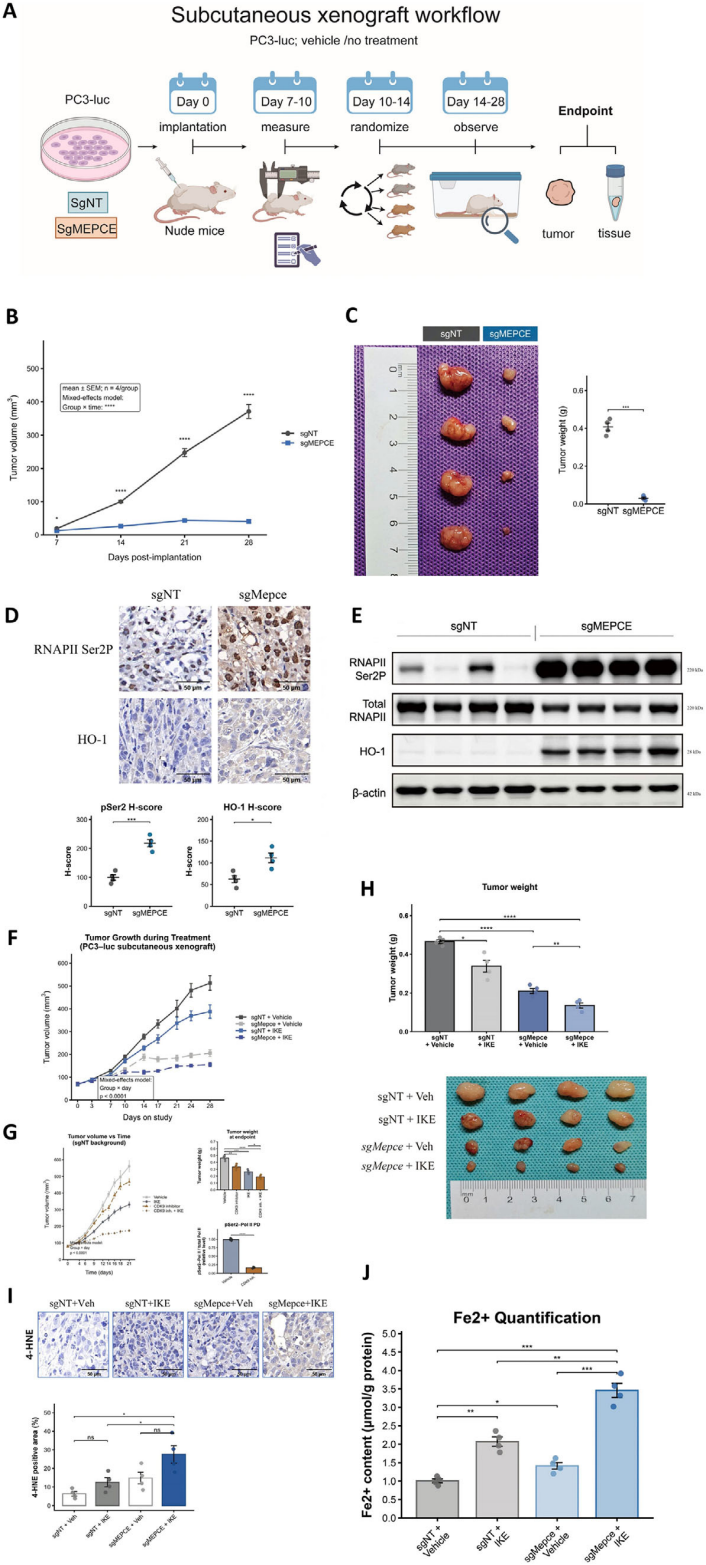
MEPCE loss restrains tumor fitness and amplifies ferroptosis‐linked therapeutic response in PC3 xenografts. (a) Schematic of the PC3‐luc subcutaneous xenograft workflow, including implantation, randomization, treatment/observation and endpoint collection. (b) Tumor growth curves under vehicle conditions comparing sgNT and sgMEPCE xenografts. (c) Representative endpoint tumors and quantification of tumor weight and/or tumor volume in sgNT and sgMEPCE xenografts. (d) Immunohistochemical staining of RNAPII Ser2P and HO‐1 in sgNT and sgMEPCE xenografts with corresponding quantification. (e) Immunoblot analysis of pSer2‐RNAPII, total RNAPII and HO‐1 in individually indexed xenograft tumors from sgNT and sgMEPCE groups. (f) Tumor growth under a 2 × 2 design comparing sgNT or sgMEPCE tumors treated with vehicle or IKE. (g) Complementary intervention arm in sgNT tumors treated with vehicle, IKE, CDK9 inhibitor, or CDK9 inhibitor plus IKE, with tumor‐growth and endpoint‐weight readouts. (h) Endpoint tumor weights across the principal treatment groups. (i) Pharmacodynamic assessment of lipid peroxidation in PC3‐luc xenografts. Representative 4‐HNE immunohistochemistry and quantification of 4‐HNE‐positive area are shown for sgNT or sgMEPCE tumors treated with vehicle or IKE. (j) Quantification of intratumoral ferrous iron in PC3‐luc xenografts from the indicated treatment groups. Fe^2+^ content was measured in tumor lysates and normalized to total protein concentration. Data are presented as nmol Fe2+ per mg protein. Significance is indicated by asterisks: ns, not significant; ^*^ *p* < 0.05; ^**^ *p* < 0.01; ^***^ *p* < 0.001; ^****^ *p* < 0.0001.

To verify that downstream pharmacodynamic changes occurred in the context of sustained target suppression, we examined MEPCE expression in tumor tissues by immunohistochemistry. In PC3‐luc xenografts, sgMEPCE tumors showed markedly reduced MEPCE staining relative to sgNT controls, and MEPCE remained low in sgMEPCE tumors irrespective of vehicle or IKE treatment (Figure S3a). These findings support the interpretation that the differences in pSer2‐RNAPII, HO‐1, lipid peroxidation and iron‐handling markers in the xenograft setting occurred in tumors with sustained MEPCE suppression rather than reflecting a secondary effect of drug exposure on target abundance. To further evaluate the ferroptosis‐related tissue state, we examined GPX4 expression and glutathione‐related parameters. In PC3 xenografts, IKE treatment was associated with reduced GPX4 signal, with the weakest staining observed in sgMEPCE tumors exposed to IKE (Figure S3e). Consistently, IKE decreased reduced GSH levels, increased GSSG abundance and markedly decreased the GSH/GSSG ratio, with the strongest effect in the combined sgMepce plus IKE group (Figure S4a).

Tumor tissue analyses supported in vivo activation of this pathway following MEPCE suppression. Immunohistochemical staining revealed stronger nuclear pSer2‐RNAPII signal in sgMEPCE tumors than in sgNT controls, together with increased HO‐1 staining; both changes were supported by blinded semi‐quantitative scoring (Figure 5d). Immunoblot analysis of individually indexed tumors recapitulated these findings, showing increased pSer2 relative to total RNAPII and increased HO‐1 abundance in sgMEPCE tumors, indicating that pathway activation was reproducible across biological replicates (Figure 5e). Thus, tumor‐intrinsic MEPCE suppression was associated in vivo with activation of an elongation‐linked RNAPII mark together with increased HMOX1/HO‐1 output, providing a rationale to test whether this state modulates response to a ferroptosis‐linked therapeutic strategy.

MEPCE suppression potentiated antitumor drug response and increased depth of response, with pharmacodynamic changes consistent with enhanced lipid peroxidation and iron stress. In a 2 × 2 design, both sgMEPCE and IKE treatment individually constrained tumor growth relative to sgNT vehicle controls, whereas the combination produced the most pronounced suppression of tumor growth (Figure 5f). In a complementary intervention arm using sgNT tumors, pharmacological CDK9 inhibition, alone and in combination with IKE, also reduced tumor growth and endpoint tumor weight, together with on‐target suppression of pSer2‐RNAPII consistent with modulation of the elongation axis (Figure 5g). Across the four principal groups, endpoint tumor weights were lowest in the sgMEPCE plus IKE condition, consistent with representative tumor images (Figure 5h). MEPCE suppression potentiated antitumor response to IKE and was accompanied by biochemical evidence of iron‐dependent lipid peroxidation in vivo. In the 2 × 2 xenograft treatment design, IKE treatment increased 4‐HNE staining in sgNT tumors, whereas sgMEPCE tumors showed a higher basal lipid‐peroxidation state and the strongest 4‐HNE signal after IKE exposure (Figure 5i). Quantification of 4‐HNE‐positive area followed the expected graded pattern, with the lowest signal in sgNT vehicle tumors, intermediate signals in the single‐perturbation groups, and the highest signal in the sgMEPCE plus IKE group. An early pharmacodynamic sampling window further showed increased 4‐HNE positivity in sgMEPCE plus IKE tumors compared with sgNT plus IKE tumors, supporting detection of lipid‐peroxidation changes before terminal tissue effects dominate the endpoint readout (Figure 5i). To directly test whether this lipid‐peroxidation phenotype was associated with iron accumulation, we quantified intratumoral ferrous iron. Fe2+ content was increased by IKE treatment and was also elevated in sgMEPCE tumors under vehicle conditions, consistent with an iron‐primed state following MEPCE loss. The highest Fe2+ burden was observed in sgMEPCE tumors treated with IKE (Figure 5j).

### 2.6 Inducible Mepce Suppression Potentiates Ferroptosis‐linked Tumor Control With Early PD Capture in an Immunocompetent Model

We next established an immunocompetent syngeneic framework to determine whether tumor‐intrinsic Mepce suppression amplifies response to IKE while permitting both early pharmacodynamic sampling and end‐of‐treatment tissue analyses. The experimental design is outlined in Figure 6a and includes RM‐1 tumor implantation, stratified randomization, doxycycline‐induced activation of shMepce after tumor establishment, a defined treatment window with vehicle or a ferroptosis‐inducing regimen, an early pharmacodynamic collection point, and end‐of‐treatment harvesting for molecular and histological analyses. Under vehicle conditions, doxycycline‐induced Mepce suppression reduced tumor size relative to shNT controls, and serial growth measurements confirmed slower tumor growth throughout the observation period, indicating impaired tumor fitness in the immunocompetent setting (Figure 6b,c).

**FIGURE 6:**
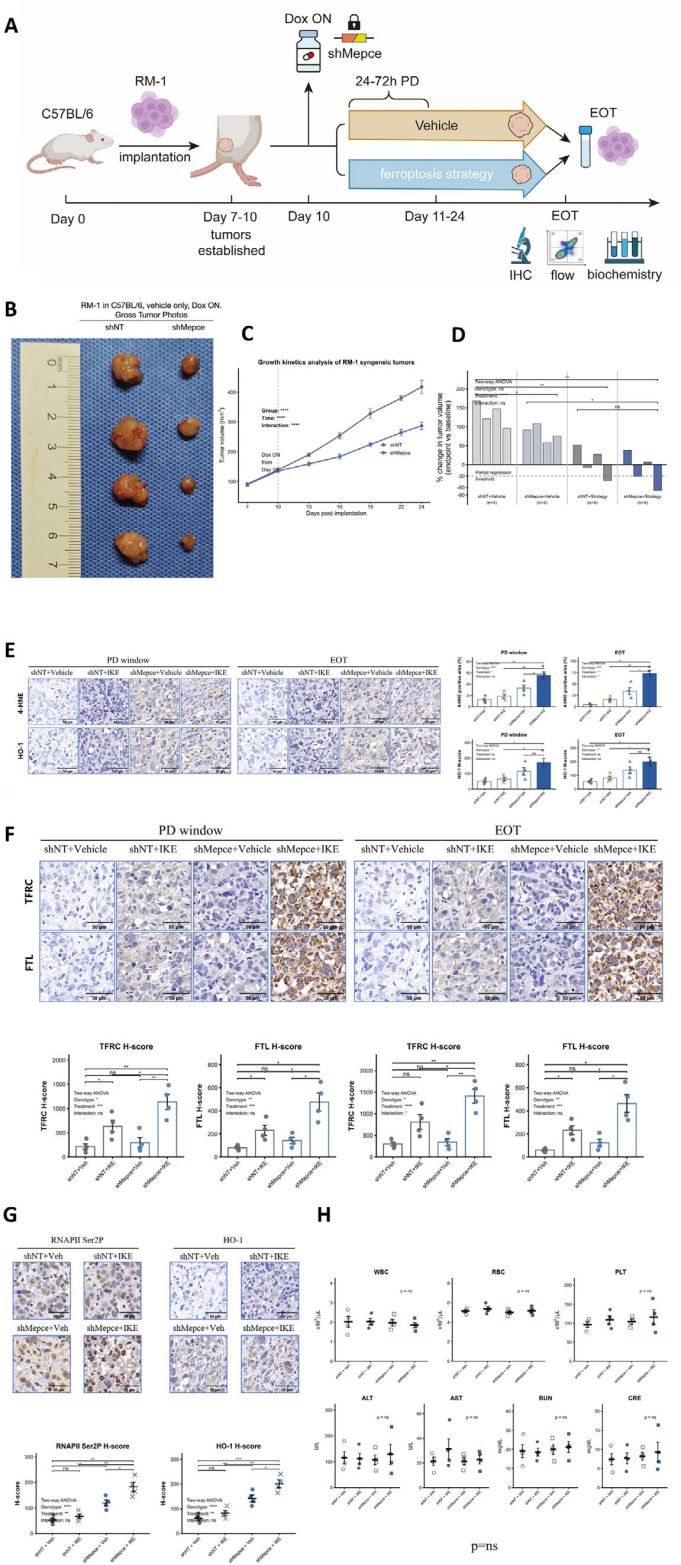
Inducible Mepce suppression potentiates ferroptosis‐linked tumor control with early pharmacodynamic capture in an immunocompetent RM‐1 model. (a) Schematic of the RM‐1 syngeneic workflow, including implantation, Dox‐inducible Mepce suppression, treatment window, early pharmacodynamic collection and end‐of‐treatment harvesting. (b) Representative endpoint tumors under vehicle conditions comparing shNT and shMepce groups. (c) Tumor growth curves after doxycycline induction of shMepce or control constructs. (d) Waterfall analysis of tumor‐volume change under the 2 × 2 design, showing response depth and partial regression events in the indicated groups. (e) Pharmacodynamic immunohistochemistry for 4‐HNE and HO‐1 at the early pharmacodynamic window and end of treatment, with corresponding quantification. (f) Iron‐handling and iron‐burden readouts, including TFRC, ferritin‐related markers and Perls staining, with quantitative analysis. (g) Tolerability assessment including body weight over time, complete blood count and serum biochemistry measurements. (h) Immunohistochemical assessment of nuclear RNAPII Ser2P and HO‐1 in RM‐1 tumors under the indicated conditions, with quantification. Data are presented as mean ± s.e.m. unless otherwise indicated. Statistical tests are described in Methods. Significance is indicated by asterisks: ns, not significant; ^*^ *p* < 0.05; ^**^ *p* < 0.01; ^***^ *p* < 0.001; ^****^ *p* < 0.0001.

We next examined Mepce expression in the RM‐1 syngeneic model to determine whether inducible target suppression persisted across the treatment course. At both the early pharmacodynamic window and the end‐of‐treatment time point, shMepce tumors displayed markedly weaker Mepce staining than shNT tumors, whereas IKE treatment alone did not produce a major reduction in Mepce staining in the shNT background (Figure S3b, c). These data support the interpretation that the associated changes in 4‐HNE, HO‐1, TFRC, ferritin‐related markers, iron burden and RNAPII Ser2 phosphorylation were linked to sustained tumor‐intrinsic Mepce suppression. Consistent with enhanced ferroptotic stress, GPX4 signal was reduced after IKE treatment and showed further attenuation in shMepce tumors under IKE exposure (Figure S3f). Likewise, IKE decreased reduced GSH, increased GSSG, and lowered the GSH/GSSG ratio, with the strongest effect observed in shMepce tumors exposed to IKE (Figure S4b).

Mepce suppression enhanced response depth to the ferroptosis‐inducing strategy and strengthened early pharmacodynamic signals consistent with ferroptotic stress. In a 2 × 2 analysis of tumor‐volume change, the ferroptosis‐inducing regimen suppressed tumor growth in the control background, whereas its combination with Mepce suppression shifted responses further toward tumor shrinkage and increased the fraction of deep responders, including partial regression events defined by the threshold in the panel (Figure 6d). Pharmacodynamic immunohistochemistry supported engagement of the proposed mechanism at both the early sampling window and the end‐of‐treatment time point: 4‐HNE and HO‐1 signals were strongest in the combined shMepce plus treatment condition, and 4‐HNE was already elevated at the early pharmacodynamic collection point (Figure 6e). Markers of iron‐handling stress, including TFRC and ferritin species, were likewise increased in the same direction, and IHC staining indicated increased intratumoral iron burden, consistent with an iron‐dependent lipid peroxidation state rather than nonspecific injury (Figure 6f).

Ferroptosis‐ and iron‐directed rescue partially reversed tumor control and reduced lipid‐peroxidation pharmacodynamics, while preserving overall tolerability and mechanistic consistency within tumors. Complete blood count and serum biochemistry measurements did not show overt adverse changes in the directions displayed, supporting a tolerability window for the treatment conditions used (Figure 6g). Consistent with persistence of the proposed transcriptional mechanism in the immunocompetent setting, tumor sections showed increased nuclear RNAPII Ser2 phosphorylation and increased HO‐1 in the Mepce‐suppressed background, with further enhancement under IKE conditions in the indicated comparisons (Figure 6h). Together, these findings support a model in which tumor‐intrinsic Mepce suppression cooperates with ferroptosis induction in vivo, enhances iron‐dependent lipid peroxidation, and remains mechanistically linked to the RNAPII Ser2P–HO‐1 stress axis in an immunocompetent context.

Mepce knockdown accelerates ferroptotic death through a CDK9–NRF2–Hmox1 axis that amplifies iron and lipid oxidation Mepce knockdown synergized with IKE in RM‐1 cells in a manner consistent with ferroptosis and iron dependence. In a 2 × 2 design, relative growth measurements showed that Mepce knockdown and IKE each suppressed cell growth, whereas the combination produced a greater effect than either perturbation alone (Figure 7a). To support the interpretation of the ex vivo slice experiments, we further assessed Mepce expression in the corresponding source tumors from which slices were generated. Tumors derived from the shMepce condition showed substantially reduced Mepce staining compared with shNT‐derived tumors, confirming that the slice assays were performed on tumors that retained the expected target‐suppressed state before ex vivo processing (Figure S3d). At the RM‐1 end‐of‐treatment time point, GPX4 staining followed the same directional pattern (Figure S3g). In the ex vivo setting, glutathione measurements showed reduced GSH, increased GSSG and the lowest GSH/GSSG ratio in the shMepce plus IKE condition, consistent with reduced antioxidant buffering capacity (Figure S4c).

**FIGURE 7:**
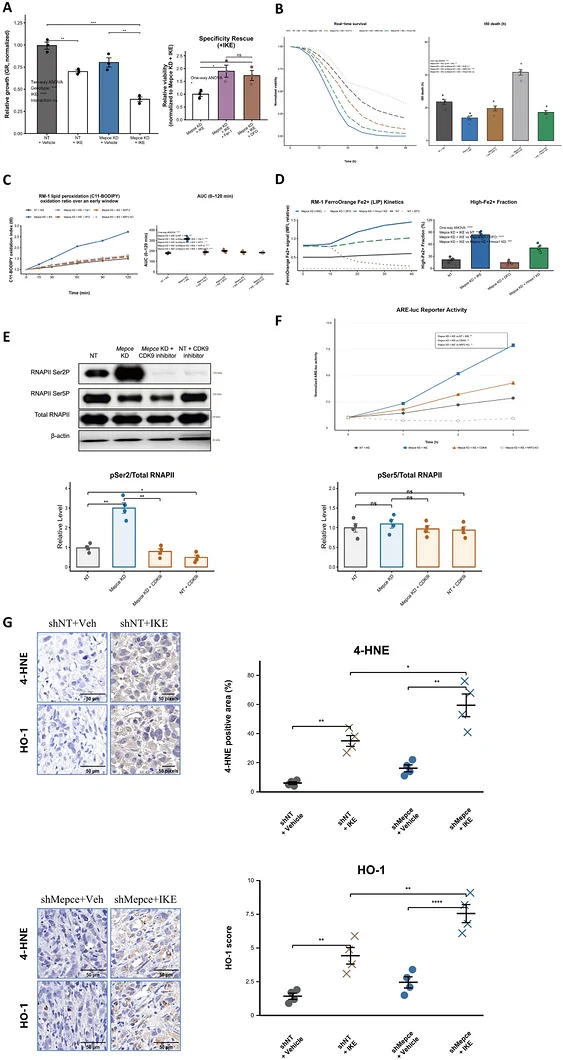
RM‐1 cell assays and ex vivo tumor slices link Mepce loss to accelerated ferroptotic stress under IKE. (a) Relative‐growth analysis of NT and Mepce‐knockdown RM‐1 cells under vehicle or IKE. (b) Real‐time survival kinetics of NT and Mepce‐knockdown RM‐1 cells treated with IKE. (c) Early lipid‐peroxidation kinetics measured by C11‐BODIPY in NT and Mepce‐knockdown RM‐1 cells treated with IKE, with Ferrostatin‐1, DFO, CDK9 inhibition, or NRF2 knockout; summary metric, AUC of the oxidation ratio over time. (d) Labile Fe^2+^ kinetics measured by FerroOrange in NT and Mepce‐knockdown RM‐1 cells treated with IKE, with DFO or Hmox1 knockdown; summary metrics, fluorescence intensity over time and high‐Fe2+ fraction. (e) Immunoblot of RNAPII Ser2P, RNAPII Ser5P, and total RNAPII in NT and Mepce‐knockdown RM‐1 cells with or without CDK9 inhibition; summary metric, phospho‐RNAPII/total RNAPII ratio. (f) NRF2 nuclear activation by immunofluorescence and ARE‐luciferase kinetics in NT and Mepce‐knockdown RM‐1 cells under the indicated perturbations; summary metrics, nuclear/cytoplasmic NRF2 ratio and normalized firefly/Renilla luciferase signal. (g) Ex vivo RM‐1 tumor slices derived from NT or doxycycline‐induced Mepce‐knockdown tumors treated with vehicle, IKE; readouts, 4‐HNE and HO‐1 by IHC. Summary metrics, positive area and histologic score, as indicated for each marker. Significance is indicated by asterisks: ns, not significant; ^*^ *p* < 0.05; ^**^ *p* < 0.01; ^***^ *p* < 0.001; ^****^ *p* < 0.0001.

Time‐resolved survival measurements showed that Mepce knockdown advances death kinetics under IKE and that this acceleration is constrained by CDK9, NRF2 and Hmox1. Real‐time viability curves revealed earlier and more rapid loss of viability in Mepce‐knockdown cells exposed to IKE than in matched control cells, consistent with a lowered death threshold (Figure 7b). Inhibition of CDK9, loss of NRF2, or suppression of Hmox1 shifted these trajectories toward delayed death relative to the Mepce‐knockdown plus IKE condition, with the corresponding kinetic summary metric changing in the same direction (Figure 7b). These reversals support a model in which a CDK9‐linked transcriptional program and an NRF2–Hmox1 gate actively contribute to accelerated ferroptotic death.

Mepce knockdown accelerated early lipid peroxidation and labile Fe^2+^ accumulation, and these effects were constrained by upstream pathway perturbation. Early C11‐BODIPY measurements showed that Mepce knockdown under IKE produced faster and stronger lipid oxidation than the corresponding control condition, and upstream pathway perturbation with ferroptosis suppression, iron chelation, CDK9 inhibition, or NRF2 loss reduced this early oxidative burden in the indicated comparisons (Figure 7c). FerroOrange time courses similarly showed that Mepce knockdown under IKE increased both the magnitude and rate of Fe^2+^ accumulation, whereas iron chelation suppressed this signal and Hmox1 knockdown reduced Fe^2+^ accumulation in the same direction (Figure 7d). These findings place labile iron expansion and lipid oxidation as early, pathway‐linked chemical outputs that track closely with the accelerated death kinetics observed under Mepce knockdown plus ferroptotic stress.

Mepce knockdown also increased RNAPII Ser2 phosphorylation and NRF2/ARE activation in a CDK9‐ and NRF2‐dependent manner. Immunoblotting showed increased RNAPII Ser2 phosphorylation in Mepce‐knockdown cells relative to controls, whereas CDK9 inhibition reduced this elevation; by contrast, changes in RNAPII Ser5 phosphorylation were less prominent (Figure 7e). In parallel, NRF2 activation and ARE‐luciferase output were increased under Mepce knockdown, suppressed by CDK9 inhibition, and largely abolished by NRF2 deletion, thereby establishing NRF2 dependence of the readout (Figure 7f). Together, these data link Mepce knockdown to a CDK9‐dependent elongation state accompanied by enhanced NRF2 activity, providing a mechanistic bridge to accelerated iron accumulation and lipid peroxidation kinetics.

Ex vivo tumor slices recapitulated the ferroptosis‐linked pharmacodynamic signals and their rescue, indicating that the amplified chemical endpoints are tumor‐intrinsic. Tumor slices generated from control or Mepce‐knockdown tumors showed increased 4‐HNE and HO‐1 signals following ex vivo exposure to IKE, with stronger induction in slices derived from Mepce‐knockdown tumors under the same treatment condition (Figure 7g). Together, these findings indicate that the amplified chemical pharmacodynamics associated with Mepce loss are retained outside the systemic context and are therefore largely tumor‐intrinsic.

## 3 Discussion

This study defines a tumor‐intrinsic transcriptional gate that shapes ferroptosis vulnerability in prostate cancer models by linking pause control to iron‐dependent lipid peroxidation. Using genome‐wide CRISPR screens under graded GPX4 inhibition, we nominated MEPCE loss as a conserved sensitizer across PC3 and DU145 backgrounds and then established mechanistic and in vivo relevance through perturbation and rescue logic. The work resolves a key ambiguity in ferroptosis biology—whether upstream transcriptional regulation can be placed causally before the chemical endpoints that execute ferroptotic death—by connecting an elongation‐licensing axis to early labile iron and lipid peroxidation readouts in cells and tumors. The findings further indicate that this axis is not restricted to cell culture, but persists in xenografts and immunocompetent syngeneic tumors where therapeutic interaction, pharmacodynamics and immune context can be evaluated together (Figure 8). In prostate cancer, this question is particularly relevant because tumor progression and treatment resistance are increasingly understood as manifestations of adaptive cell‐state remodeling rather than of fixed oncogenic circuitry alone [20]. Advanced prostate tumors can persist under chronic therapeutic, metabolic and oxidative stress by reprogramming redox buffering, lipid handling and survival pathways, thereby creating marked heterogeneity in ferroptosis sensitivity across models and disease states [21, 22, 23]. Within this context, our findings support the view that ferroptotic vulnerability may be governed not only by terminal antioxidant effectors, but also by upstream transcriptional states that determine how rapidly and how strongly stress‐adaptive programs are deployed [24]. This framing may be especially pertinent in aggressive or therapy‐refractory prostate cancer settings, where stress adaptation and lineage plasticity have emerged as central features of tumor persistence and phenotypic diversification [25, 26].

Mechanistically, our data support a stepwise model in which MEPCE maintains the inhibitory 7SK snRNP state to constrain P‐TEFb, and MEPCE loss destabilizes RN7SK and weakens HEXIM1–P‐TEFb association, consistent with release of CDK9 activity. In both global and locus‐resolved assays, MEPCE loss increases RNAPII Ser2 phosphorylation in a CDK9‐sensitive manner and shifts nascent transcription patterns toward reduced promoter‐proximal pausing with increased gene‐body transcription, including at stress‐responsive loci. This elongation‐licensing state accelerates NRF2/ARE output and establishes HMOX1 as a kinetic bottleneck, supported by reporter kinetics, nascent RNA time courses and cis‐element dependence, and constrained by CDK9 inhibition and NRF2 perturbation. Downstream, the pathway converges on ferroptosis‐consistent chemical endpoints—accelerated labile Fe^2+^ accumulation and lipid peroxidation—that are reduced by iron chelation and ferroptosis suppressors and are also attenuated by perturbing CDK9, NRF2 or Hmox1, linking the transcriptional gate to execution‐phase chemistry. Rescue experiments provide the strongest causal support at the level of ferroptosis modality and upstream gating, whereas occupancy‐based distinctions at HMOX1 suggest context‐dependent modes in which elongation licensing can dominate even when NRF2 occupancy changes are limited.

These findings align with prior work establishing ferroptosis as an iron‐dependent, lipid peroxidation–driven death program and with evidence that NRF2‐dependent transcription can remodel redox and iron‐handling states in cancer cells [27]. They also fit with broader models in which promoter‐proximal pausing and P‐TEFb‐mediated elongation licensing determine the speed and amplitude of inducible transcriptional programs [28]. The present work extends these frameworks by placing a 7SK‐associated upstream regulator, MEPCE, before a ferroptosis‐relevant stress module and by emphasizing kinetics—rather than only steady‐state expression—as a determinant of death‐threshold crossing [29]. At the same time, the data support a nuanced view of NRF2 control at HMOX1: increased NRF2 nuclear activation and ARE dependence are clear, but locus occupancy differences are not uniformly required, leaving room for competing models in which elongation licensing acts downstream of relatively stable occupancy to amplify output. More generally, our in vivo results are consistent with the idea that ferroptosis‐inducing strategies can be potentiated by tumor state, and they suggest that transcriptional gating can be one such state variable that is measurable by PD markers in tumors. More broadly, these findings extend emerging concepts in transcription biology by suggesting that promoter‐proximal pausing is not merely a generic regulator of inducible gene expression, but can function as a phenotypically meaningful gate for cell‐death susceptibility. Previous studies have established that the 7SK snRNP and P‐TEFb axis shape the timing and amplitude of rapid transcriptional responses to stress and differentiation cues [30, 31, 32]. The present work places this framework in the context of ferroptosis and suggests that altered pause release can influence whether stress‐responsive transcription remains adaptive or instead crosses a threshold that promotes iron accumulation and lipid peroxidation. In this sense, MEPCE may be viewed less as an isolated ferroptosis factor than as part of a higher‐order transcriptional control layer that tunes death‐threshold crossing through elongation kinetics.

A point that warrants careful interpretation is the relationship between HMOX1 and the broader NRF2‐responsive transcriptional program engaged after MEPCE loss. Our data strongly support HMOX1 as a functionally important downstream effector, particularly because its accelerated induction is kinetically prominent and its perturbation attenuates labile iron accumulation, lipid peroxidation, and ferroptosis sensitivity. At the same time, the transcriptional and pathway‐level analyses indicate that pause‐control remodeling is not restricted to HMOX1 alone, but rather amplifies a broader NRF2/ARE‐associated stress program that includes additional antioxidant and redox‐regulatory targets. We therefore interpret HMOX1 not as the sole consequence of elongation remodeling, but as a particularly prominent gate‐like node within a wider NRF2‐driven transcriptional landscape, one whose kinetic positioning makes it especially influential for ferroptosis‐relevant iron and lipid chemistry.

Several limitations should be considered when interpreting these findings. First, the current study is centered on prostate cancer models (PC3, DU145, and RM‐1), and the extent to which this pause‐control framework generalizes across tumor lineages, genetic contexts, and microenvironmental states remains to be determined. Second, although multiple perturbation and rescue approaches support ferroptosis involvement, some pharmacologic inhibitors and rescue agents may have pleiotropic effects; accordingly, additional orthogonal genetic epistasis at ferroptosis‐relevant nodes would further refine pathway assignment and constrain alternative interpretations. Third, the mechanistic link to 7SK snRNP destabilization and P‐TEFb release is supported by RN7SK stability analyses and co‐immunoprecipitation, but direct structural analysis or genome‐wide mapping of complex redistribution was not performed in the present study. Finally, although the revised manuscript now specifies IKE as the ferroptosis‐inducing agent used in vivo, the extent to which the observed interaction will generalize across mechanistically distinct ferroptosis‐inducing modalities remains to be established.

Despite these limitations, the work has practical implications for ferroptosis‐oriented therapeutic design and for interpreting tumor PD responses. The data suggest that targeting a transcriptional gating node can reshape the kinetics of NRF2/HMOX1 output and thereby modulate iron and lipid oxidation dynamics that determine whether ferroptosis thresholds are crossed, offering a mechanistically grounded route to expand therapeutic windows. In vivo, the concordance of pathway markers (RNAPII Ser2P, HO‐1) with lipid peroxidation and iron‐handling PD supports a PD suite that could be used to monitor on‐pathway engagement in tumors, including within early sampling windows (Figure 8). A concrete next step is to perform tumor‐intrinsic epistasis in vivo by combining Mepce suppression with enforced blockade of key downstream nodes to test necessity for tumor response and PD shifts.

**FIGURE 8:**
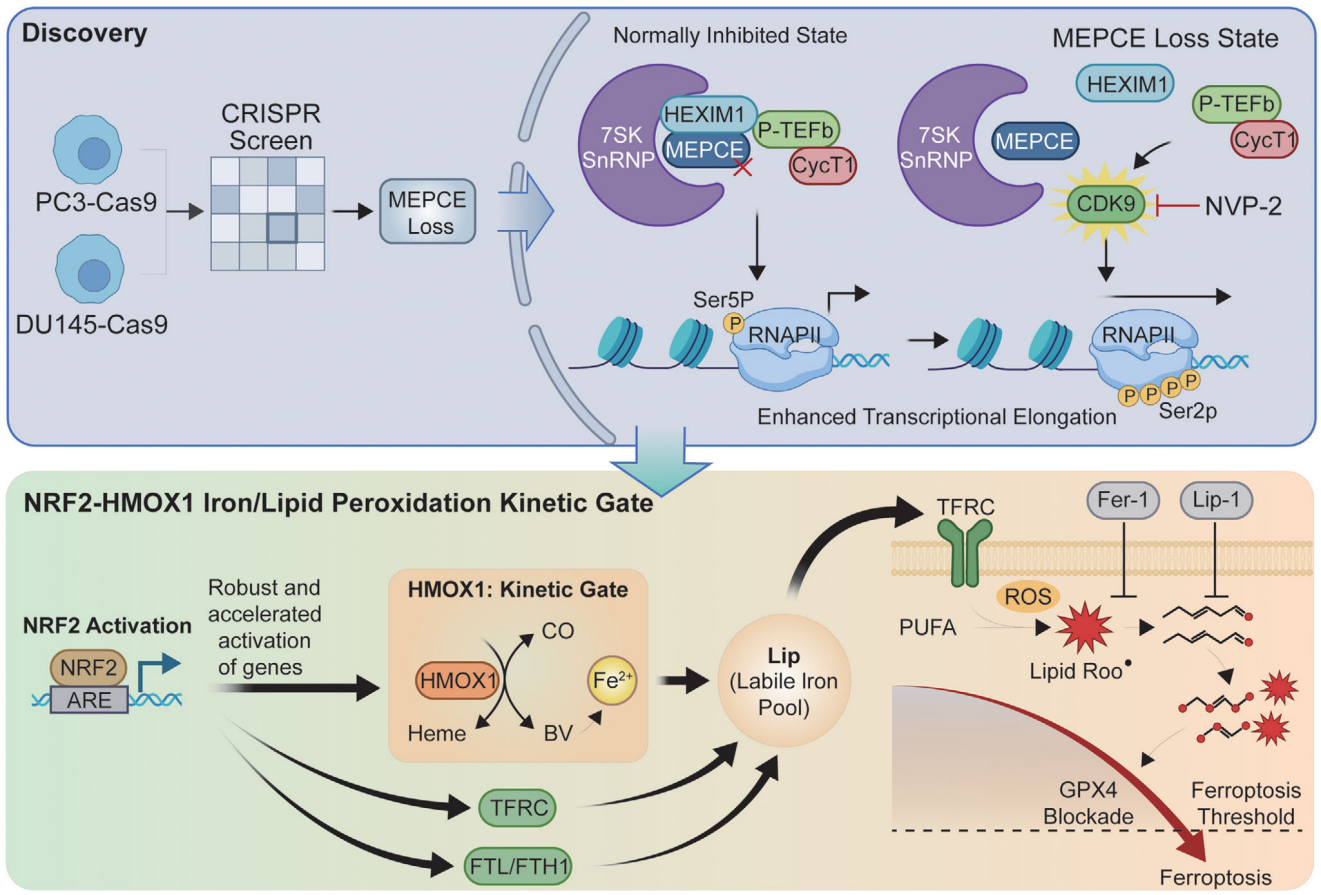
Proposed model of how MEPCE loss lowers the ferroptosis threshold through CDK9‐dependent pause‐control remodeling and NRF2‐responsive stress transcription.

## 4 Method

### 4.1 Cell Culture

Human prostate cancer cell lines PC3‐Cas9 and DU145‐Cas9 and the murine prostate cancer cell line RM‐1 were maintained in RPMI 1640 supplemented with 10% fetal bovine serum and 1% penicillin–streptomycin at 37°C in a humidified incubator with 5% CO_2_. This culture condition is consistent with prior prostate‐cancer ferroptosis studies using PC3 and DU145 cells.

### 4.2 Lentiviral Transduction and Genetic Perturbations

For pooled CRISPR screening, a lentiviral genome‐wide sgRNA library was packaged in HEK293T cells. PC3‐Cas9 and DU145‐Cas9 cells were transduced at a low multiplicity of infection (MOI ∼0.3) to favor single‐guide integration per cell. Twenty‐four hours after infection, medium was replaced, and infected cells were selected with puromycin for 3 days. After selection, cells were expanded for several additional days before T0 genomic DNA collection and treatment initiation.

For targeted validation experiments, individual sgRNAs were cloned into lentiviral sgRNA vectors. At least two independent sgRNAs were used per target gene. A non‐targeting sgRNA served as control. For MEPCE rescue experiments, an sgRNA‐resistant MEPCE cDNA was re‐expressed in the corresponding knockout background using a lentiviral expression construct. For the RM‐1 system, doxycycline‐inducible shMepce or matched control constructs were introduced before implantation.

### 4.3 Genome‐Wide Pooled CRISPR Screening

Genome‐wide pooled CRISPR knockout screens were performed in PC3‐Cas9 and DU145‐Cas9 cells under DMSO control, sublethal GPX4 inhibition (RSL3_low), lethal GPX4 inhibition (RSL3_high), and ferroptosis‐specific rescue conditions. After library transduction, puromycin selection, and recovery, cells were split into the indicated treatment arms while maintaining at least 300–500‐fold representation per sgRNA throughout the experiment, a standard range for pooled negative‐selection CRISPR screening. Baseline genomic DNA was collected at T0 after selection and recovery. Endpoint genomic DNA was harvested after 14–21 days of selection. sgRNA cassettes were PCR‐amplified and subjected to next‐generation sequencing, followed by gene‐level scoring using MAGeCK.

### 4.4 Small‐Molecule Treatments

RSL3 was used as the GPX4‐directed ferroptosis inducer in cell‐based assays. Ferrostatin‐1 (Fer‐1), Liproxstatin‐1 (Lip‐1), and deferoxamine (DFO) were used as rescue reagents. NVP‐2 was used as the CDK9 inhibitor. Actinomycin D was used for transcriptional shutoff experiments. For most rescue experiments, cells were pretreated with rescue compounds or inhibitors for 60 min before addition of RSL3. DMSO concentration was matched across treatment groups. A representative configuration consistent with the present study is as follows: Ferrostatin‐1, 10 µm; Liproxstatin‐1, 0.5 µm; DFO, 100 µm; NVP‐2, 50 nm; actinomycin D, 5 µg/mL.

### 4.5 Cell Viability and Dose–Response Assays

For endpoint viability assays, cells were seeded in 96‐well plates at 2000–5000 cells per well and allowed to adhere overnight. The next day, cells were treated with RSL3 over the indicated concentration range with or without rescue compounds, followed by CellTiter‐Glo measurement according to the manufacturer's instructions. Luminescence values were normalized to matched vehicle‐treated controls. Dose–response curves were fitted using a four‐parameter logistic model in GraphPad Prism, and IC50 values or area under the curve (AUC) were calculated as indicated.

### 4.6 Real‐Time Survival and Death Assays

For kinetic survival assays, cells were plated in imaging‐compatible plates and treated as indicated. Images were acquired at regular intervals using a live‐cell imaging platform, and the fraction of dead cells was quantified over time. Kinetic parameters including time‐to‐onset, time‐to‐half‐maximal response (t50), maximal slope, and AUC were derived from fitted time‐course data.

### 4.7 RT–qPCR

Total RNA was extracted using TRIzol, and cDNA was synthesized from 0.5–1 µg RNA using a reverse transcription kit. qPCR was performed using SYBR Green chemistry with gene‐specific primers for MEPCE, RN7SK, HMOX1, NQO1, TXNRD1, GAPDH, and other indicated targets. Relative expression was calculated using the ΔΔCt method normalized to GAPDH or U6, as appropriate.

### 4.8 Actinomycin D Chase Assay

To assess transcript stability, cells were pretreated as indicated and then exposed to actinomycin D to block de novo transcription. RNA was collected at serial time points after actinomycin D addition, and HMOX1 mRNA abundance was quantified by RT–qPCR. Transcript levels were normalized to the value at time 0, and decay curves were generated to compare relative transcript stability between conditions.

### 4.9 Nascent RNA Measurements

Nascent‐transcription analyses in this study were performed using two explicitly defined approaches. For gene‐specific nascent RNA time‐course measurements, newly synthesized RNA was quantified by a qPCR‐based nascent RNA capture assay. Cells were treated under the indicated conditions and labelled with the same nascent‐RNA capture system throughout the study according to the manufacturer's protocol. Total RNA was isolated at the indicated time points, and the nascent RNA fraction was enriched before reverse transcription. qPCR was then performed for HMOX1, NQO1, TXNRD1, and the control transcript GAPDH using SYBR Green chemistry. Relative nascent transcript abundance was normalized to the indicated control condition or time 0 sample, as specified in the figure panels.

For genome‐wide pausing and locus‐level nascent transcription analyses, a sequencing‐based nascent transcription profiling approach was used. Cells were processed under matched perturbation conditions, and nascent RNA libraries were prepared using the same workflow across all samples. Libraries were sequenced on an Illumina platform and aligned to the appropriate reference genome. Genome‐wide analyses were then used to derive metagene profiles, promoter‐proximal pausing‐index distributions, and locus‐level transcription tracks for stress‐responsive genes, including HMOX1, NQO1 and TXNRD1.

### 4.10 ARE‐Luciferase and HMOX1 Reporter Assays

For ARE‐luciferase assays, cells were seeded in 96‐well plates and transfected with an ARE‐responsive firefly luciferase reporter together with a Renilla luciferase construct for normalization. After 24 h, cells were treated as indicated and collected at serial time points. Firefly luciferase values were normalized to Renilla luciferase values within each well and then to the relevant control condition.

For HMOX1 reporter assays, cells were transfected with a reporter containing wild‐type HMOX1 regulatory elements or the corresponding ARE‐mutant construct. Reporter signals were collected over time after treatment. For kinetic analyses, curves were fitted to derive parameters including t_onset, t50, and maximal slope.

### 4.11 Immunoblotting

Whole‐cell lysates were prepared in RIPA buffer supplemented with protease and phosphatase inhibitors. Protein concentrations were determined using a BCA assay. Equal amounts of protein (20 µg per lane) were resolved by SDS–PAGE and transferred to PVDF membranes. Membranes were blocked in 5% milk and incubated with primary antibodies and the indicated loading controls. HRP‐conjugated secondary antibodies and chemiluminescent detection were used for visualization.

### 4.12 Co‐Immunoprecipitation

For co‐immunoprecipitation, clarified lysates containing 1000 µg total protein were incubated with target‐specific antibodies or matched IgG controls overnight at 4°C, followed by capture using Protein A/G agarose beads. Beads were washed multiple times with lysis buffer, and bound proteins were eluted in SDS sample buffer and analyzed by immunoblotting.

### 4.13 Tumor Ferrous Iron Quantification

Fresh or snap‐frozen tumor tissues were weighed and homogenized on ice in iron‐assay buffer using a tissue homogenizer. Homogenates were centrifuged at high speed at 4°C to remove insoluble debris, and the supernatants were collected for ferrous iron measurement. Fe^2+^ content was quantified using a ferrous iron colorimetric/fluorometric assay according to the manufacturer's instructions.

### 4.14 Lipid Peroxidation Assay

Lipid peroxidation was measured using C11‐BODIPY 581/591. Cells were incubated with 2 µm C11‐BODIPY for 30 min at 37°C in the dark, washed, and analyzed by flow cytometry. Oxidation was quantified as the shift from reduced to oxidized fluorescence or as the ratio of oxidized to reduced signal.

### 4.15 Labile Iron Measurement

Labile Fe^2+^ was measured using FerroOrange. Cells were incubated with 1 µm FerroOrange for 30 min at 37°C in the dark, washed, and analyzed by flow cytometry. Mean fluorescence intensity and the fraction of high‐Fe^2+^ cells were quantified using FlowJo.

### 4.16 Immunofluorescence

For immunofluorescence, cells grown on coverslips were fixed in 4% paraformaldehyde for 15 min, permeabilized with 0.1 Triton X‐100, blocked in 5% BSA, and incubated with primary antibodies overnight at 4°C. After incubation with fluorophore‐conjugated secondary antibodies, nuclei were counterstained with DAPI. Images were acquired under identical exposure settings within each experiment. Nuclear/cytoplasmic NRF2 ratios and nuclear pSer2‐RNAPII intensities were quantified using ImageJ.

### 4.17 smFISH

smFISH was performed using a probe‐based fluorescence in situ hybridization protocol directed against HMOX1 transcripts. Cells were fixed, permeabilized, hybridized with fluorescent probes according to the manufacturer's protocol, and imaged by confocal microscopy. Quantified outputs included puncta per cell and the fraction of cells containing active transcription sites.

### 4.18 RNA‐Seq and Transcriptomic Analysis

Total RNA from biologically independent replicate samples was subjected to library preparation using a poly(A)‐selection workflow. Sequencing was performed on an Illumina platform, and reads were aligned to the appropriate reference genome. Differential expression analysis was performed using a standard pipeline. Genes were ranked by log2 fold change and adjusted *p* value.

### 4.19 ChIP–qPCR

All occupancy analyses at HMOX1 regulatory regions and gene‐body positions in this study were performed using ChIP–qPCR. Cells were cultured under the indicated treatment conditions and crosslinked with 1% formaldehyde at room temperature for 10 min, followed by quenching with 125 mm glycine. Chromatin was isolated and sonicated to generate DNA fragments of approximately 200–500 bp. Equal amounts of sheared chromatin were incubated overnight at 4°C with antibodies against NRF2, RNA polymerase II Ser2P, or CDK9, together with matched IgG controls. Immune complexes were captured using Protein A/G magnetic beads, washed sequentially under low‐salt, high‐salt, LiCl and TE conditions, and then eluted. Crosslinks were reversed, and DNA was purified for qPCR analysis.

qPCR primers were designed to amplify HMOX1 promoter‐proximal regions, distal ARE‐containing regulatory elements, and gene‐body regions, as indicated in the corresponding figures. Enrichment was calculated relative to input DNA, with IgG or a negative control region used as the background control where appropriate.

### 4.20 Animal Studies

PC3‐luc subcutaneous xenografts were established with tumor cells carrying sgNT or sgMEPCE, followed by tumor measurement over time and endpoint tissue collection for IHC and immunoblotting. Volume‐balanced randomization was performed after tumors became measurable, followed by a vehicle/no treatment observation window. A separate arm evaluated vehicle, IKE, CDK9 inhibitor, and CDK9 inhibitor plus IKE in the sgNT background, with pSer2‐RNAPII used as a PD readout. RM‐1 syngeneic subcutaneous tumors were established in immunocompetent C57BL/6 mice, randomized after tumor establishment, and subjected to Dox‐on Mepce suppression and vehicle or a ferroptosis‐inducing strategy. Early PD windows (48 h) and end‐of‐treatment harvesting were used for mechanistic tissue analyses, and rescue interventions were tested for in vivo reversibility. Tolerability was assessed by longitudinal body weight and endpoint blood measures. This study was reviewed and approved by the Experimental Animal Ethics Committee of Shanghai University of Traditional Chinese Medicine. The ethics approval number was PZSHUTCM2503240003.

### 4.21 IKE Treatment In Vivo

Imidazole ketone erastin (IKE), an in vivo‐suitable ferroptosis inducer, was used as the ferroptosis‐inducing agent in this study. IKE was freshly prepared in vehicle consisting of 5% DMSO and 95% HBSS (pH 4), sterilized through a 0.22‐µm filter, and administered by intraperitoneal injection at 23 mg/kg once daily, as indicated in the treatment schematics. Vehicle‐treated mice received the corresponding volume of 5% DMSO/95% HBSS. Treatment was initiated after tumors reached the predefined size for randomization and continued for the indicated treatment window or until endpoint collection. This dosing strategy was selected on the basis of prior studies establishing IKE as a pharmacologically tractable ferroptosis inducer for in vivo tumor models.

### 4.22 Histology and Immunohistochemistry

Tumors were fixed in 10% neutral‐buffered formalin, embedded in paraffin, sectioned at 5 µm, and processed for hematoxylin and eosin staining or immunohistochemistry. Antigen retrieval was performed using citrate buffer (pH 6.0) or EDTA buffer (pH 9.0) according to antibody optimization. Sections were incubated with antibodies against MEPCE/Mepce, RNAPII Ser2P, HO‐1, 4‐HNE, GPX4, ferritin‐related markers, and other indicated targets, followed by HRP‐conjugated secondary detection and DAB development. Perls staining was performed according to standard procedures. Quantification was performed using H‐score, positive area, or positive nuclei fraction, as appropriate.

### 4.23 Human Ethics Approval and Clinical Specimens

Human prostate tumor specimens used in this study were collected with approval from the Ethics Committee of Shuguang Hospital affiliated to Shanghai University of Traditional Chinese Medicine. The study was conducted in accordance with the Declaration of Helsinki and relevant institutional guidelines. The ethics approval number was 2022‐1210‐147‐02. Where applicable, written informed consent was obtained from all participants prior to sample collection. Human tissue samples were used only for research purposes and were de‐identified before analysis.

### 4.24 Glutathione Measurements

Snap‐frozen tumor tissue or ex vivo tumor slices were homogenized in assay buffer on ice, and total GSH, reduced GSH, oxidized glutathione (GSSG), and the GSH/GSSG ratio were measured using a commercial glutathione assay kit according to the manufacturer's instructions. Values were normalized to total protein or tissue weight.

### 4.25 Statistical Analysis

Data are presented as mean ± s.e.m. unless otherwise indicated. Statistical analyses were performed using GraphPad Prism and/or R. Two‐group comparisons were performed using unpaired two‐tailed Student's *t*‐tests. Multi‐group comparisons were analyzed by one‐way or two‐way ANOVA with appropriate post–hoc multiple‐comparison correction. Time‐course and repeated‐measures data were analyzed using repeated‐measures two‐way ANOVA.

## Author Contributions

**Tongtong Zhang**: conceptualization, investigation, writing – original draft, writing – review and editing, visualization, validation, funding acquisition. **Mingyue Tan**: conceptualization, data curation, resources, supervision. **Xiangyang Zhan**: methodology, validation, visualization, writing – original draft, funding acquisition. **Chuanmin Chu**: conceptualization. **Huirong Zhu**: validation, conceptualization, supervision. **Dongliang Xu**: formal analysis, visualization, validation, investigation, conceptualization, funding acquisition, supervision. **Jiexiang Zhang**: software, formal analysis, project administration, conceptualization, funding acquisition. **Jianyi Gu**: conceptualization. **Xinyu Zhai**: methodology, project administration. **Guanqun Ju**: validation.

## Funding

Shanghai University of Traditional Chinese Medicine Affiliated Shuguang Hospital Siming Fund (SGKJ‐202502).

## Conflicts of Interest

The authors declare no conflict of interest.

## Supporting information




**Supporting File**: advs76958‐sup‐0001‐SuppMat.docx.

## Data Availability

The data that support the findings of this study are available from the corresponding author upon reasonable request.
