# Supplementary material for: Mepce Restrains Ferroptosis in Prostate Cancer Through 7SK–P‐TEFb Pause Control
Source: Adv Sci (Weinh). 2026 Aug 5;13(60):e76958. doi: 10.1002/advs.76958 (PMC13440031; doi:10.1002/advs.76958)
Supplement: Supplementary file 1 [file ADVS-13-e76958-s001.docx]

**Supplementary Figure S1**

**Supplementary Figure S1 | MEPCE expression across clinicopathological strata and its representative relationship to RNAPII Ser2 phosphorylation in prostate cancer.**

a–c, MEPCE expression in the displayed TCGA prostate cancer strata, including pathologic T stage (a), pathologic N stage (b), and clinical M stage (c), with normal samples plotted as shown.

d, Immunoblot analysis of six individually indexed human prostate tumor specimens (p1–p6) for MEPCE and Ser2-phosphorylated RNA polymerase II (pSer2-RNAPII); β-actin served as the loading control.

**Supplementary Figure S2**

**Supplementary Figure S2 | Targeted validation of additional shared screening hits identified in the genome-wide CRISPR screen.**

a,b, RT–qPCR validation of BRPF3 disruption in DU145-Cas9 (a) and PC3-Cas9 (b) cells using independent sgRNAs.

c,d, RT–qPCR validation of KAT6A disruption in DU145-Cas9 (c) and PC3-Cas9 (d) cells.

e,f, RT–qPCR validation of PHF12 disruption in DU145-Cas9 (e) and PC3-Cas9 (f) cells.

g,h, RT–qPCR validation of PHF23 disruption in DU145-Cas9 (g) and PC3-Cas9 (h) cells.

i,j, RSL3 dose–response viability curves showing partial resistance after BRPF3 disruption in DU145-Cas9 (i) and PC3-Cas9 (j) cells.

k,l, RSL3 dose–response viability curves showing increased sensitivity after KAT6A disruption in DU145-Cas9 (k) and PC3-Cas9 (l) cells.

m,n, RSL3 dose–response viability curves showing increased sensitivity after PHF12 disruption in DU145-Cas9 (m) and PC3-Cas9 (n) cells.

o,p, RSL3 dose–response viability curves showing increased sensitivity after PHF23 disruption in DU145-Cas9 (o) and PC3-Cas9 (p) cells.

**Supplementary Figure S3**

**Supplementary Figure S3 | Target suppression and GPX4-associated tissue state across PC3 xenografts and RM-1 tumors.**

a, Immunohistochemical staining of MEPCE in PC3 xenografts across sgNT/sgMEPCE and vehicle/IKE conditions, showing maintained target suppression in vivo.

b,c, Immunohistochemical staining of Mepce in RM-1 syngeneic tumors at the early pharmacodynamic window (b) and end-of-treatment time point (c), showing sustained inducible target suppression.

d, Mepce staining in the source tumors used for ex vivo slice experiments, comparing shNT and shMepce conditions before ex vivo processing.

e, GPX4 immunohistochemistry in PC3 xenografts under vehicle or IKE, with the weakest staining in the sgMEPCE plus IKE condition.

f,g, GPX4 immunohistochemistry in RM-1 syngeneic tumors at the early pharmacodynamic window (f) and end-of-treatment time point (g) under the indicated vehicle/IKE conditions. Representative images are shown. Scale bars, 50 μm.

**Supplementary Figure S4**

**Supplementary Figure S4 | Glutathione-system disruption is enhanced by MEPCE/Mepce suppression across xenograft, syngeneic, and ex vivo settings.**

a, Reduced GSH, GSSG, and the GSH/GSSG ratio in PC3 xenografts under sgNT/sgMEPCE and vehicle/IKE conditions.

b, Reduced GSH, GSSG, and the GSH/GSSG ratio in RM-1 syngeneic tumors at the early pharmacodynamic window under shNT/shMepce and vehicle/IKE conditions.

c, Total GSH, reduced GSH, GSSG, and the GSH/GSSG ratio in ex vivo RM-1 tumor slices under vehicle, IKE, and Ferrostatin-1 (Fer-1) rescue conditions. Four plotted observations per group are shown.
